# Trends in the availability and prices of quality-assured tuberculosis drugs: a systematic analysis of Global Drug Facility Product Catalogs from 2001 to 2024

**DOI:** 10.1186/s12992-024-01047-7

**Published:** 2024-06-25

**Authors:** Stefan Kohler, Jay Achar, Christiaan Mulder, Norman Sitali, Nicolas Paul

**Affiliations:** 1 Heidelberg Institute of Global Health, Faculty of Medicine and University Hospital, Heidelberg University, Heidelberg, Germany; 2 Institute of Social Medicine, Epidemiology and Health Economics, Charité – Universitätsmedizin Berlin, corporate member of Freie Universität Berlin and Humboldt-Universität zu Berlin, Berlin, Germany; 3 Department of Global Public Health, Karolinska Institutet, Stockholm, Sweden; 4 KNCV Tuberculosis Foundation, The Hague, The Netherlands; 5 Médecins Sans Frontières, Berlin, Germany

**Keywords:** Cost analysis, Drug costs, Drug supply, Global Drug Facility, Price trends, Tuberculosis drugs, Tuberculosis

## Abstract

**Background:**

The Global Drug Facility (GDF) of the Stop TB Partnership was launched in 2001 with the goal of increasing access to quality-assured tuberculosis (TB) drugs and products. We aimed to describe the TB drugs and prices available from the GDF over time and to assess trends.

**Methods:**

We searched the internet, including an internet archive, for past and recent GDF Product Catalogs and extracted the listed TB drugs and prices. We calculated the lowest price for the most common drug formulations assuming drugs with similar active pharmaceutical ingredients (APIs) are substitutes for each other. We assessed time trends in the TB drugs and prices offered by the GDF in univariable regressions over the longest possible period.

**Results:**

We identified 43 different GDF Product Catalogs published between November 2001 and May 2024. These product catalogs included 122 single medicines (31 APIs), 28 fixed-dose combinations (9 API combinations), and 8 patient kits (8 API regimens and other materials). The number of TB drugs listed in the GDF Product Catalog increased from 9 (8 APIs) to 55 (32 APIs). The price decreased for 17, increased for 19, and showed no trend for 12 APIs. The price of 15 (53.6%) of 28 APIs used against drug-resistant TB decreased, including the price of drugs used in new treatment regimens. The decreasing price trend was strongest for linezolid (-16.60 [95% CI: -26.35 to -6.85] percentage points [pp] per year), bedaquiline (-12.61 [95% CI: -18.00 to -7.22] pp per year), cycloserine (-11.20 [95% CI: -17.40 to -4.99] pp per year), pretomanid (-10.47 [95% CI: -15.06 to -5.89] pp per year), and rifapentine (-10.46 [95% CI: -12.86 to -8.06] pp per year). The prices of 16 (61.5%) of 23 APIs for standard drug-susceptible TB treatment increased, including rifampicin (23.70 [95% CI: 18.48 to 28.92] pp per year), isoniazid (20.95 [95% CI: 18.96 to 22.95] pp per year), ethambutol (9.85 [95% CI: 8.83 to 10.88] pp per year), and fixed-dose combinations thereof.

**Conclusions:**

The number of TB drugs available from the GDF has substantially increased during its first 23 years of operation. The prices of most APIs for new TB treatments decreased or remained stable. The prices of most APIs for standard drug-sensitive TB treatment increased.

**Supplementary Information:**

The online version contains supplementary material available at 10.1186/s12992-024-01047-7.

## Introduction

With 1.3 million deaths in 2022, an estimated incidence of 10.6 million in 2022, and estimated donor support of $2.46 billion in 2023, tuberculosis (TB) remains a major global public health concern [1, 2]. Resistance against first-line TB drugs is common and includes mono-resistant, poly-resistant, multidrug-resistant (MDR), and extensively drug-resistant (XDR) TB. Drug-resistant TB usually requires longer treatment with more drugs or newer TB drugs than the treatment of drug-susceptible TB. Drug-related costs are a major contributor to TB treatment costs, constituting, for instance, the largest or second largest cost component in cost-effectiveness analyses of MDR TB treatment in Peru, Estonia, Russia, and the Philippines [3]. In cost analyses of drug-susceptible TB treatment, TB drugs constituted 25% of overall treatment costs in low-income countries [4]. In addition to high drug costs, TB programs face issues of substandard and counterfeit TB drugs. An analysis from pharmacies in 17 low-income and middle-income countries showed that 9.1% of the analyzed rifampicin and isoniazid samples failed to meet basic quality targets [5]. Use of substandard and counterfeit TB drugs may cause adverse drug effects, TB treatment failure, aggravation of antimicrobial resistance, loss of confidence in national TB programs, and elevated costs for people with TB and TB programs [6].

To increase access to low-priced quality-assured TB drugs, the Global Drug Facility (GDF) was founded by the Stop TB Partnership in 2001 [7, 8]. The GDF provides procurement and supply services for TB drugs and diagnostics; it uses competitive bidding to pursue bulk purchases from TB drugs suppliers [7, 9, 10]. To ensure that the TB drugs available from the GDF meet World Health Organization (WHO) quality standards, the GDF established quality assurance standards and control systems [11]. Suppliers to the GDF generally need to provide drugs that are prequalified by the WHO or authorized by a stringent regulatory authority [12]. The GDF also aims at shaping the supply markets for TB products by increasing demand and decreasing risks for suppliers, by encouraging new manufacturers to enter the market, by facilitating innovation, and by lowering transaction costs [10]. The GDF’s market share varies across WHO regions [13] and was globally estimated at 42.9% for second-line tuberculosis drugs and 19.2% for first-line TB drugs in 2012 [14].

GDF products and prices are relevant for numerous national TB programs and are used in cost and cost-effectiveness analyses of TB care [15–21]. Despite the common use of GDF prices as reference prices, past GDF Product Catalogs, GDF products, or GDF prices have been neither comprehensively assembled nor systematically analyzed in the existing literature. Relatedly, the development of the availability and prices of TB drugs from the GDF over time has not been studied. We aimed to describe TB drugs and prices listed in the GDF Product Catalog from the launch of the GDF in 2001 until May 2024 and to assess time trends in the TB drugs and prices offered by the GDF.

## Methods

### Study design

First, we systematically searched for past GDF Product Catalogs and extracted their content. Second, we analyzed the TB drugs and prices listed in the GDF Product Catalogs for trends over the longest possible period.﻿ The reporting of information followed the Guidelines for Accurate and Transparent Health Estimates Reporting (GATHER) [22].

### Global Drug Facility Product Catalog

The GDF Product Catalog is a list of TB products available from the GDF for TB treatment in adults and children. In recent years, the GDF has separately published a ‘GDF Medicines Catalog’ and a ‘GDF Diagnostics, Medical Devices & Other Health Products Catalog’. Products are listed at their current purchase prices in US Dollar ($) unless noted otherwise. A unique price or a price range is provided in the catalog for most products. For some products, the prices and/or availability are only available upon request from the GDF. Discounts or donations are indicated where available (e.g., the Janssen-USAID Bedaquiline Donation Program [23]). Early GDF Product Catalogs were mostly published as a website. Later product catalogs were published as a PDF brochure on the GDF website. As the GDF Product Catalog was updated, past catalogs were no longer available through the GDF Product Catalog website [24].

### Global Drug Facility product codes

The GDF uses product codes to uniquely identify TB drugs and other products. The product code of single medicines represents a combination of the drug abbreviation, dosage in milligram or gram, drug packaging, and the number of drug units per pack. The drug packaging can be either ampoule (A), bottle (BTL), blistered tablets or capsules (B), blistered dispersible tablets (B-DT), loose tablets or capsules in a jar, container, or bottle (L), vial (V), or sachet (S) (e.g., Rbt-150-(L)-100 for a jar with 100 capsules containing 150 mg rifabutin). Product codes of fixed-dose combinations (FDCs) can contain an additional prefix with the number of active ingredients in the drug (e.g., 4-FDC/RHZE-150/75/400/275-(B)-672 for a blister with 672 tablets combining rifampicin 150 mg, isoniazid 75 mg, pyrazinamide 400 mg, and ethambutol 275 mg). The product code of patient kits (PKs) combines a prefix and patient kit identifier (e.g., PK-Cat I & III-A). Drug and patient kit abbreviations used within the product code and patient kit contents are described in the supplementary Tables S1–S3.

### Search strategy

We searched the internet archive ‘Wayback Machine’ (https://archive.org/) from inception to May 21, 2024, for five URLs that had been used to reference GDF Product Catalogs in publications or archived versions of the GDF website (https://web.archive.org/web/*/stoptb.org/gdf/drugsupply/drugs.available.html, https://web.archive.org/web/*/stoptb.org/gdf/drugsupply/drugs_available.asp, https://web.archive.org/web/*/stoptb.org/gdf/drugsupply/drugs_diagnostics.asp, https://web.archive.org/web/*/stoptb.org/gdf/drugsupply/product_catalog.asp, https://web.archive.org/web/*/stoptb.org/global-drug-facility-gdf/gdf-product-catalog). We usually screened four archived website copies per year during the period the respective URL was active to identify changes in the GDF Product Catalog. When possible, we screened the first archived copy in the first two quarters of each year and the last archived copy in the last two quarters. We included catalogs in this study that showed product or price updates. To identify GDF Product Catalog updates, we screened archived websites for changes in the displayed publication date, products, and prices and/or for changes in linked PDFs and embedded thumbnails of catalogs. We added GDF Product Catalogs to the internet archive search results that we had either previously downloaded from the GDF website and archived or that we identified through internet searches via Google or on the GDF website. The internet searches included the terms: Global Drug Facility, GDF, product catalogue, product catalog, medicines catalogue, and medicines catalog.

### Data extraction

From the GDF Product Catalogs included in the study, we included single medicines, FDCs, and patient kits for adults and children. We excluded TB drugs for special purpose (e.g., GDF India Programme products) and non-pharmaceutical TB products if listed (e.g., syringes, water for injection, diagnostic supplies, or adherence technologies). For the single medicines, FDCs, and patient kits in each GDF Product Catalog, we extracted the product code, active pharmaceutical ingredient(s) (API[s]), drug dose, drug packaging, drug units per pack, and the price per pack. From catalogs published in 2018 or later, we also extracted drug usage information (drug-susceptible TB, drug-resistant TB, and TB preventive treatment that was also called treatment of latent TB infection). When no publication date was listed for a website update, we used the archiving date. When no publication month was listed for a PDF catalog, we extracted the creation date from the PDF-file’s metadata.

### Data analysis

First, we summarize the product codes, which describe the API(s), dose, form, and units per pack, for all TB drugs listed in the identified GDF Product Catalogs. We further describe the number of times a drug was listed in the GDF Product Catalog﻿, the number of listing changes, the last price at which a drug was available, the first and last year in which a TB drug was listed, and the years of the lowest and highest drug price. Second, we describe and assess how the number of drug products and APIs available from the GDF changed over time, grouping single medicines, FDCs, and patient kits. Third, we describe and assess how the price of the APIs of the TB drugs listed in the GDF Product Catalog developed over time based on calculating the lowest price-per-dose across TB drugs with the same API(s). We use GDF product codes and drug abbreviations when reporting results.

To assess linear time trends, we regressed the number of drugs and the nominal drug prices listed in the GDF Product Catalog on the publication date of the catalog from which data were extracted. The publication month was converted to a fraction of a year. We estimated univariable regression models with Huber-White robust standard errors and assessed trends for the longest observational period of each outcome. We also estimated the same regression models with drug prices adjusted to a January 2024 price level using the gross domestic product deflator for advanced economies from the International Monetary Fund (IMF) World Economic Outlook database [25]. We interpolated linearly between annual deflator values to obtain monthly deflator values (supplementary Fig. S1). For the number of products listed in the GDF Product Catalog, we performed a supplementary regression analysis comparing the fit of models with a linear time trend and a stepwise increase. When a GDF Product Catalog reported a price range, we used the lowest price in the regression analysis. Where drugs with the same API, API combination, or API regimen were listed in a product catalog in different doses, we calculated the price equivalent of the most listed drug for the regression analysis using the lowest price per dose of APIs available from the GDF in different pharmaceutical forms. For instance, different rifampicin (R) formulations like R-150-(B)-800 and R-300-(B)-100 were treated as perfect substitutes of each other.

Discounts listed in the GDF Product Catalog for certain orders (e.g., 20% free goods for each 10 packs of Bdq-100-(L)-188 ordered between 2020 and 2024) were included in the calculation of the lowest price. From April 2015 to March 2019, bedaquiline (Bdq) was available through the Bedaquiline Donation Program between the US Agency for International Development (USAID) and Janssen Therapeutics [23]. The Bdq-100-(L)-188 price of zero listed in the 2016 catalog was excluded from the descriptive statistics and regression analysis. Drug prices that were listed as upon request/contact GDF were also excluded from the analysis. We grouped drugs that differed only in their packaging (i.e., blister/loose and vial/ampoule) and patient kits Cat-II that differed by 4 units of streptomycin and/or 4 units of syringes and needles. We report monetary price changes in US-dollar ($) and percentage point (pp) price changes to allow for trend comparisons across APIs. To estimate changes in pp, prices were normalized such that the first listed price is 100. Occasionally, drug prices were listed in Euro or CHF. These prices were converted to US-Dollar using the IMF annual exchange rate for the respective years [26]. Statistical significance was assumed at *P* < 0.05. All analyses were performed in Stata 18.5 SE.

## Results

### Search results

**Fig. 1:**
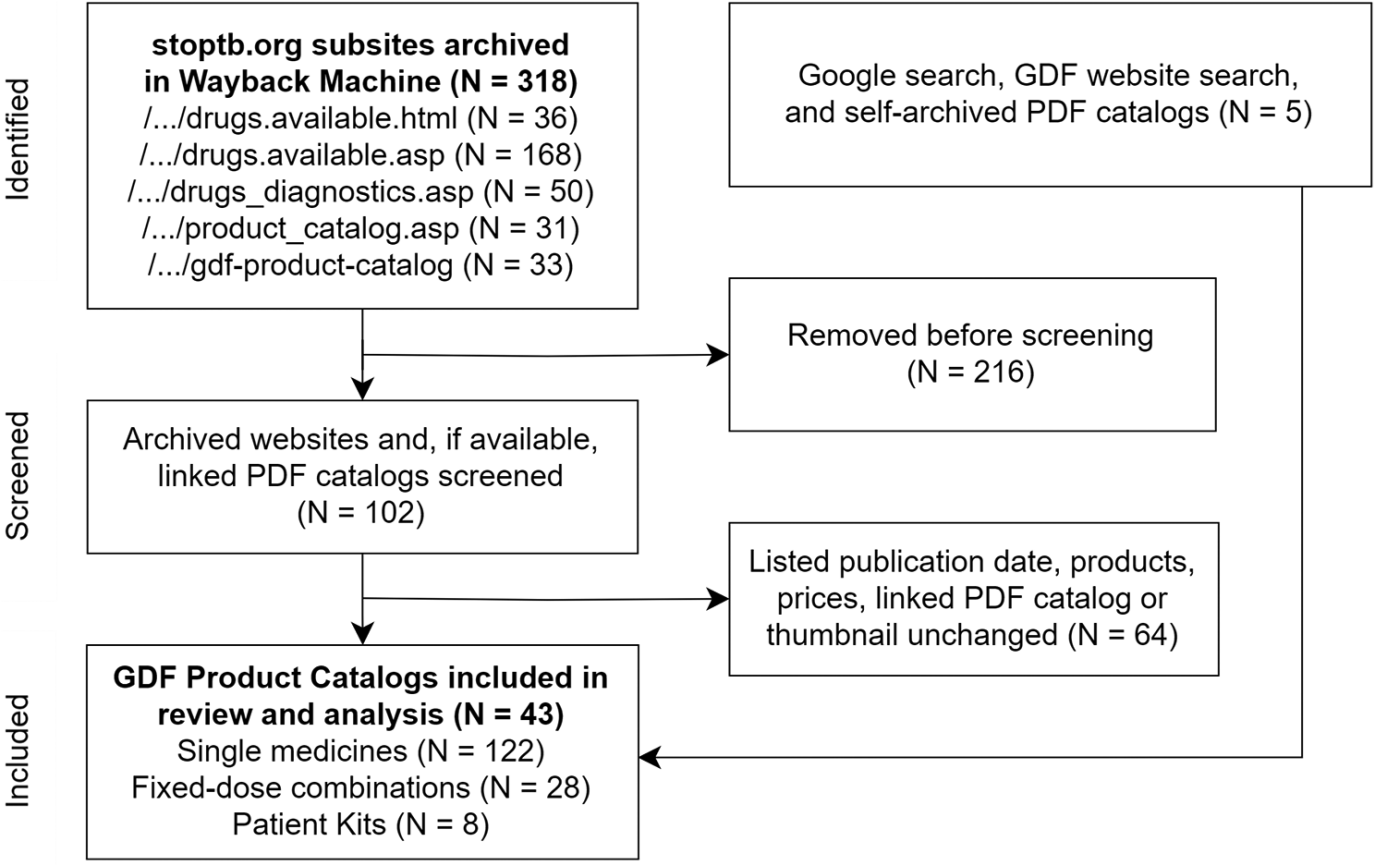
Flow chart of GDF Product Catalogs identified, screened, and included in the study

We identified 318 copies of GDF Product Catalog subsites in the internet archive ‘Wayback Machine’. Based on screening 102 archived copies for changes in the listed publication date, products, prices, linked PDF catalog, or thumbnail image of a linked PDF catalog, we included 38 GDF Product Catalogs in the study. An additional 5 GDF Product Catalogs were added to the study from Google searches, GDF website searches, and self-archived PDF catalogs. Product catalogs were published as a website between 2001 and 2011 and as downloadable PDF brochures between 2011 and 2024. A ‘GDF Medicines Catalog’ and a ‘GDF Diagnostics, Medical Devices & Other Health Products Catalog’ were published as PDF brochures since 2016. No GDF Product Catalog updates could be found for the years 2012, 2013, 2015, and 2017. In other years, the number of catalog updates identified ranged from 1 to 6 per year (Fig. 1 and supplementary Table S4).

### TB drugs available from the GDF

From 2001 to 2024, 122 single medicines, 28 FDCs, and 8 patient kits were listed in the GDF Product Catalog. Of these 158 drug products, 69 (43.7%) could be used against drug-susceptible TB, 127 (80.4%) against drug-resistant TB, and 21 (13.3%) for TB preventive treatment. The single medicines included 31 different APIs. The FDCs included 9 different API combinations. Patient kits were available for first-time treatment of drug-susceptible TB (Category I & III) and for recurrent TB treatment (Category II). The patient kits included 8 different API regimens and differed further by whether auto-disabling syringes were included or not (e.g., PK-Cat II-A1 versus II-A2).

Products were listed 1–42 times across the 43 GDF Product Catalogs included in the study. The median (IQR) number of products listed in the catalog was 27 (18–48) single medicines, 9 (8–11) FDCs, and 1 (1–7) patient kit. Between 2001 and 2024, we observed 413 changes in 1362 listings of single medicines, 176 changes in 431 listings of FDCs, and 57 changes in 117 listings of patient kits including listings of product prices or availability only upon request from the GDF (Table 1 and supplementary Tables S4, S5).

**Table 1:** Tuberculosis drugs and prices in the Global Drug Facility Product Catalog, 2001–2024

**Product code**	Price listings	Listings	Changes	Last price ($)	Price range ($)	**Period available**	Year		Use		
							Lowest price	Highest price	DS TB	DR TB	TPT
**Single medicines (122 drugs)**											
Am-500-(A)-1	1	1	0	1.00		2014	2014	2014		✓	
Am-500-(V)-5	1	1	0	8.25		2014	2014	2014		✓	
Am-500-(V)-20	1	5	1	49.47		2009–2011	2009	2009		✓	
Am-500-(V/A)-10	20	24	10	6.51	6.21–15.33	2007–2023	2019	2008		✓	
Am-500-(V/A)-100	14	14	4	57.20	57.20–61.89	2016–2024	2022	2016		✓	
Amx/Clv-250/125-(B)-14	4	4	1	1.63	1.63–2.92	2014–2018	2018	2014		✓	
Amx/Clv-250/125-(B)-20	5	5	2	1.99	1.99–2.70	2014–2018	2018	2014		✓	
Amx/Clv-125/31.25-(BTL)-1	4	4	1	1.21	1.21–1.77	2016–2018	2018	2016		✓	
Amx/Clv-250/62.5-(BTL)-1	2	2	1	1.86–2.42	1.86–2.42	2014–2016	2016	2016		✓	
Amx/Clv-500/125-(B)-15	5	5	1	1.75	1.75–2.21	2014–2018	2016	2014		✓	
Amx/Clv-500/125-(B)-20	1	1	0	2.70–3.00	2.70–3.00	2014	2014	2014		✓	
Amx/Clv-500/125-(B)-21	1	1	0	2.63–3.42	2.63–3.42	2016	2016	2016		✓	
Amx/Clv-500/125-(B)-100	21	21	7	9.25–16.97	9.25–19.21	2014–2024	2021	2014		✓	
Amx/Clv-875/125-(B)-12	5	5	1	1.69	1.69–1.89	2014–2018	2016	2014		✓	
Amx/Clv-875/125-(B)-14	1	1	0	4.84		2011	2011	2011		✓	
Amx/Clv-875/125-(B)-16	1	1	0	2.16–2.88	2.16–2.88	2016	2016	2016		✓	
Amx/Clv-875/125-(B)-100	8	8	3	11.80	4.41–22.90	2014–2020	2014	2014		✓	
Bdq-20-(L)-60	15	15	0	25.53	25.53–25.53	2020–2024	2020	2024		✓	
Bdq-100-(B)-100	4	4	1	79.50	79.50–96.97	2023–2024	2024	2023		✓	
Bdq-100-(L)-188	16	21	6	122	122–400	2014–2024	2023	2019		✓	
Cfz-50-(B/L)-100	28	28	22	35.78	35.78–64.78	2014–2024	2019	2014		✓	
Cfz-100-(B/L)-100	21	21	4	50.00–80.80	50.00–127	2014–2024	2019	2014		✓	
Cla-250-(B)-14	2	2	1	1.60–1.80	1.60–4.01	2014–2016	2016	2014		✓	
Cla-500-(B)-14	2	2	1	2.72–2.87	2.72–6.22	2014–2016	2016	2014		✓	
Cm-0.5-(V)-1	1	4	1	2.99		2014–2020	2014	2014		✓	
Cm-0.5-(V)-80	1	1	0	239		2016	2016	2016		✓	
Cm-0.75-(V)-1	1	1	0	3.99		2014	2014	2014		✓	
Cm-0.75-(V)-80	1	1	0	319		2016	2016	2016		✓	
Cm(A)-1-(V)-1	6	10	1	3.21	3.21–3.21	2007–2011	2007	2009		✓	
Cm(B)-1-(V)-1	5	5	1	1.02	1.02–1.07	2007–2008	2008	2007		✓	
Cm-1-(V)-10	3	6	1	21.70–23.35	21.70–23.35	2018–2020	2018	2018		✓	
Cm-1-(V/A)-1	6	7	4	3.31–3.85	3.31–8.00	2007–2018	2018	2011		✓	
Cs-125-(B)-100	19	19	1	45.00	42.50–45.00	2018–2024	2018	2024		✓	
Cs-250-(B)-21	1	1	0	7.43–8.09	7.43–8.09	2016	2016	2016		✓	
Cs-250-(B)-40	1	1	0	7.43–8.09	7.43–8.09	2016	2016	2016		✓	
Cs(A)-250-(B)-100	1	1	0	50.96		2007	2007	2007		✓	
Cs(B)-250-(B)-100	1	1	0	14.12		2007	2007	2007		✓	
Cs-250-(B/L)-100	29	34	14	25.00–25.40	19.25–78.14	2007–2024	2018	2011		✓	
Dlm-25-(B-DT)-48	12	12	0	85.00	85.00–85.00	2021–2024	2021	2024		✓	
Dlm-50-(B)-48	4	7	2	85.00	85.00–121	2022–2024	2023	2023		✓	
Dlm-50-(B)-672	16	16	0	1700	1700–1700	2016–2023	2016	2023		✓	
E-100-(B)-500	0	5	0	Contact GDF		2009–2011			✓	✓	
E-100-(B-DT)-100	19	19	5	20.05–21.91	20.05–25.00	2018–2024	2022	2022	✓	✓	
E-100-(B/L)-100	22	22	8	3.58	3.04–8.60	2011–2024	2016	2016	✓	✓	
E-400-(B)-672	40	42	20	26.00–27.90	8.67–27.90	2002–2024	2002	2024	✓	✓	
E-400-(L)-1000	18	21	12	21.48	10.92–22.94	2001–2011	2002	2008	✓	✓	
E-600-(B)-100	1	1	0	4.00		2016	2016	2016	✓	✓	
E-800-(B)-100	1	1	0	11.11		2016	2016	2016	✓	✓	
Eto-125-(B)-90	1	1	0	10.06		2014	2014	2014		✓	
Eto-125-(B-DT)-100	19	19	5	13.30–14.48	13.00–21.25	2018–2024	2019	2018		✓	
Eto-125-(B/L)-100	4	4	1	8.14	8.14–10.00	2016–2018	2018	2016		✓	
Eto-250-(B)-10	0	3	0	Contact GDF		2009–2010				✓	
Eto-250-(B)-90	1	1	0	5.94		2014	2014	2014		✓	
Eto-250-(B/L)-100	29	34	13	9.16	6.40–11.10	2007–2024	2016	2014		✓	
H-100-(B-DT)-100	17	17	4	8.95–9.16	8.50–10.00	2018–2024	2018	2022	✓	✓	✓
H-100-(B/L)-100	23	34	10	1.24–1.27	0.85–3.10	2007–2024	2016	2016	✓	✓	✓
H-100-(L)-1000	0	11	0	Contact GDF		2007–2011			✓	✓	✓
H-300-(B)-672	40	42	19	11.48–13.23	3.76–13.52	2002–2024	2002	2022	✓	✓	✓
H-300-(L)-1000	18	21	12	6.35	3.65–7.69	2001–2011	2002	2008	✓	✓	✓
Imp/Cls-500/500-(V)-1	1	1	0	7.32–10.64	7.32–10.64	2014	2014	2014		✓	
Imp/Cls-500/500-(V)-10	20	20	7	28.50–32.20	28.50–36.00	2016–2024	2022	2018		✓	
Km-0.5-(V)-1	0	3	0	Contact GDF		2019–2020				✓	
Km-0.5-(V)-10	1	1	0	5.50		2016	2016	2016		✓	
Km-0.5-(V)-50	4	4	0	42.50	42.50–42.50	2016–2018	2016	2018		✓	
Km-1-(V)-1	0	1	0	Contact GDF		2010				✓	
Km-1-(V)-50	11	17	10	34.00–46.00	18.58–113	2007–2020	2007	2016		✓	
Km-1-(V/A)-10	6	10	4	23.36	23.36–25.80	2011–2020	2018	2014		✓	
Lfx-100-(B-DT)-100	19	19	6	11.86–12.41	11.86–90.00	2018–2024	2022	2018		✓	
Lfx-250-(B)-100	28	32	12	2.63–2.97	2.60–8.41	2007–2024	2021	2014		✓	
Lfx-500-(B)-80	1	1	0	5.62–5.82	5.62–5.82	2014	2014	2014		✓	
Lfx-500-(B)-100	28	32	12	4.82	3.96–16.21	2007–2024	2019	2014		✓	
Lfx-750-(B)-100	20	20	5	9.52–10.25	9.52–20.50	2016–2024	2022	2018		✓	
Lzd-150-(B-DT)-100	9	9	0	27.27	27.27–27.27	2022–2024	2022	2024		✓	
Lzd-600-(B)-10	4	4	1	13.39–13.79	13.39–15.21	2016–2018	2018	2016		✓	
Lzd-600-(B)-100	19	19	5	17.03–21.40	17.03–140	2018–2024	2022	2018		✓	
Lzd-600-(B)-200	1	1	0	138		2014	2014	2014		✓	
Mfx-100-(B-DT)-100	19	19	5	19.60–19.91	19.60–85.00	2018–2024	2022	2018		✓	
Mfx-400-(B)-5	3	5	3	1.93	1.93–8.40	2010–2016	2016	2011		✓	
Mfx-400-(B)-100	21	21	7	15.00–16.00	15.00–64.51	2014–2024	2022	2014		✓	
Mrp-1-(V)-1	4	4	1	3.70	3.70–4.10	2016–2018	2018	2016		✓	
Mrp-1-(V)-10	16	16	5	33.50–34.73	32.20–34.73	2019–2024	2022	2024		✓	
Ofx-200-(B/L)-100	10	15	7	3.43	3.43–5.80	2007–2016	2016	2014		✓	
Ofx-400-(B/L)-100	6	10	5	5.71	5.71–10.00	2008–2016	2016	2014		✓	
PAS-(H)-4-(S)-30	15	20	8	40.00	40.00–64.90	2007–2020	2014	2008		✓	
PAS-(Na)-4-(S)-25	20	20	1	33.00	33.00–33.75	2016–2024	2018	2016		✓	
PAS-(Na)-4-(S)-30	4	4	0	37.20	37.20–37.20	2016–2018	2016	2018		✓	
PAS-(Na)-5.52-(S)-25	2	2	1	34.25	34.25–37.50	2011–2014	2014	2011		✓	
PAS-(Na)-9.2-(S)-30	2	2	1	34.50	34.50–43.50	2011–2014	2014	2011		✓	
PAS-(Na)-100-(L)-1	3	7	3	13.50	12.49–16.00	2009–2016	2014	2011		✓	
Pa-200-(B)-100	5	5	0	131	131–131	2023–2024	2023	2024		✓	
Pa-200-(L)-26	16	16	1	34.29	34.29–52.00	2019–2024	2022	2022		✓	
Pto-250-(B)-140	1	1	0	18.10		2014	2014	2014		✓	
Pto-250-(B/L)-100	28	33	15	8.43–9.98	8.20–16.47	2007–2024	2018	2008		✓	
Pto-250-(L)-50	1	1	0	4.07		2011	2011	2011		✓	
Pyr(B6)-10-(B)-100	7	7	0	3.00	3.00–3.00	2022–2024	2022	2024	✓	✓	✓
Pyr(B6)-50-(B)-50	19	19	3	0.66	0.66–0.70	2018–2024	2022	2022	✓	✓	✓
Pyr(B6)-100-(L)-250	19	19	4	10.75	8.25–11.55	2018–2024	2018	2022	✓	✓	✓
R-150-(B)-80	0	5	0	Contact GDF		2022–2023			✓		✓
R-150-(B)-100	20	20	6	12.85	3.70–14.85	2016–2024	2016	2018	✓		✓
R-150-(B)-800	0	1	0	Contact GDF		2022			✓		✓
R-300-(B)-40	0	6	0	Contact GDF		2022–2023			✓		✓
R-300-(B)-100	20	20	7	19.33	7.30–25.00	2016–2024	2016	2018	✓		✓
Rbt-150-(B)-24	1	1	0	12.60–30.00	12.60–30.00	2014	2014	2014	✓		✓
Rbt-150-(L)-100	20	20	4	74.17	69.86–94.53	2016–2024	2021	2020	✓		✓
Rpt-150-(B)-24	20	20	4	7.15	5.25–24.00	2016–2024	2020	2016	✓		✓
Rpt-150-(B-DT)-100	1	1	0	13.80		2024	2024	2024	✓		✓
Rpt-300-(B)-100	8	8	2	33.92	33.89–33.92	2022–2024	2022	2024	✓		✓
S-0.75-(V)-50	3	4	1	2.70	2.70–2.70	2001–2003	2002	2003		✓	
S-1-(V)-10	9	13	3	5.75	5.22–5.75	2018–2024	2018	2023		✓	
S-1-(V)-50	15	15	8	5.04	2.95–5.21	2004–2010	2005	2008		✓	
S-1-(V)-100	7	14	3	64.00	64.00–68.00	2010–2022	2014	2011		✓	
Trd-250-(B)-50	6	6	3	83.30–90.00	79.40–175	2011–2018	2014	2016		✓	
Trd-250-(B)-100	16	16	0	175	175–175	2019–2024	2019	2024		✓	
Z-150-(B-DT)-100	19	19	5	14.50–15.94	1.36–15.94	2018–2024	2018	2024	✓	✓	
Z-150-(B/L)-100	0	11	0	Contact GDF		2007–2011			✓	✓	
Z-150-(L)-1000	0	11	0	Contact GDF		2007–2011			✓	✓	
Z-400-(B)-672	40	42	16	14.00	8.70–15.80	2002–2024	2006	2016	✓	✓	
Z-400-(L)-1000	18	21	11	10.99	10.04–13.00	2001–2011	2006	2005	✓	✓	
Z-500-(B)-100	2	2	1	2.56	1.72–2.56	2016	2016	2016	✓	✓	
Z-500-(B)-120	3	3	0	6.25	6.25–6.25	2018	2018	2018	✓	✓	
Z-500-(B)-672	21	21	6	13.31–14.00	12.91–22.00	2014–2024	2018	2018	✓	✓	
Z-750-(B)-672	1	1	0	31.00		2014	2014	2014	✓	✓	
All single medicines	1187	1362	413		0.66–1700	2001–2024			34	112	18
**Fixed-dose combinations (28 drug combinations)**											
3-HP-300/300-(B)-36	14	14	2	9.99–10.96	9.99–15.00	2020–2024	2023	2022	✓		✓
EH-400/150-(B)-672	21	23	14	27.43–33.81	8.92–33.81	2002–2016	2002	2016	✓		
EH-400/150-(L)-1000	18	21	12	25	11.77–26.93	2001–2011	2002	2008	✓		
HPST-Q-TIB-(L)-30	18	18	1	2.38	1.99–2.38	2018–2024	2018	2024			✓
RH-75/50-(B)-84	20	20	7	4.41–4.75	2.41–4.75	2016–2024	2016	2024	✓		✓
RH-150/100-(B)-672	2	2	0	8.87	8.87–8.87	2002	2002	2002	✓		
RH-150/100-(L)-1000	2	3	1	11.66	11.66–11.66	2001–2002	2002	2002	✓		
RH-60/60-(B)-80	0	6	0	Contact GDF		2007–2008			✓		
RH-60/60-(B)-84	3	3	0	3	3.00–3.00	2011–2016	2011	2016	✓		
RH-60/60-(L)-1000	0	11	0	Contact GDF		2007–2011			✓		
RH-150/150-(B)-672	19	21	12	23.43	9.30–23.43	2002–2016	2002	2016	✓		
RH-150/150-(L)-1000	17	19	10	24.45	11.98–26.15	2002–2011	2002	2008	✓		
RH-60/30-(B)-84	3	11	3	1.33–1.53	1.33–4.00	2007–2016	2016	2011	✓		
RH-60/30-(B)-90	0	3	0	Contact GDF		2009–2010			✓		
RH-60/30-(L)-1000	0	6	0	Contact GDF		2007–2008			✓		
RH-150/75-(B)-336	12	12	2	15.65	11.57–15.65	2018–2022	2018	2022	✓		
RH-150/75-(B)-672	39	41	19	31.78–35.08	8.80–35.08	2002–2024	2002	2024	✓		
RH-150/75-(L)-1000	18	21	12	24.92	11.50–24.92	2001–2011	2002	2011	✓		
RH-300/150-(L)-1000	2	3	1	21.4	21.40–21.40	2001–2002	2002	2002	✓		
RHE-150/75/275-(B)-672	37	38	21	52.69–55.42	17.82–99.00	2004–2024	2004	2022	✓		
RHE-150/75/275-(L)-1000	17	18	12	44.90	23.63–77.90	2004–2014	2004	2011	✓		
RHZ-60/30/150-(B)-84	3	11	3	1.96–2.21	1.96–6.00	2007–2016	2016	2011	✓		
RHZ-60/30/150-(B)-90	0	3	0	Contact GDF		2009–2010			✓		
RHZ-60/30/150-(L)-1000	0	6	0	Contact GDF		2007–2008			✓		
RHZ-75/50/150-(B)-84	20	20	7	6.28–6.65	2.95–6.65	2016–2024	2016	2024	✓		
RHZE-150/75/400/275-(B)-336	12	12	2	30.93	23.75–30.93	2018–2022	2018	2022	✓		
RHZE-150/75/400/275-(B)-672	40	42	21	62.90–68.20	22.00–68.90	2002–2024	2002	2022	✓		
RHZE-150/75/400/275-(L)-1000	20	23	14	57.50	30.50–57.50	2001–2014	2002	2014	✓		
All fixed-dose combinations	357	431	176		1.33–99	2001–2024			27	0	3
**Patient kits (8 drug regimens)**											
PK-Cat I & III-A	37	39	19	33.51–35.10	11.56–35.10	2004–2024	2004	2024	✓		
PK-Cat I & III-B	12	14	7	30.25	22.54–32.27	2006–2014	2006	2011	✓		
PK-Cat I & III-C	12	14	7	16.59	14.26–16.70	2006–2016	2006	2008	✓		
PK-Cat II-A1	12	13	7	97.40	46.27–97.40	2006–2014	2006	2014	✓		
PK-Cat II-A2	12	13	6	52.08	41.90–54.23	2005–2010	2005	2008	✓		
PK-Cat II-B1	11	12	6	98.90	52.27–98.90	2006–2014	2006	2014	✓		
PK-Cat II-B2	10	11	5	54.21	43.13–56.66	2006–2010	2006	2008	✓		
PK-Cat II-C	1	1	0	90.40		2014	2014	2014	✓		
All patient kits	107	117	57		11.56–98.90	2004–2024			8	0	0
**All medicines (N = 158)**	1651	1910	646		0.66–1700	2001–2024			69	112	21

Lowest price = first year listed with this price. Highest price = last year listed with this price. DS = drug-susceptible, DR = drug-resistant, TPT = TB preventive treatment. (A) = ampoule, (BTL) = bottle, (B) = blister, (B-DT) = blistered dispersible tablets, (B/L) = blister or loose (L) = loose, (S) = sachet, (V) = vial, (V/A) = vial or ampoule. The product code of single medicines and fixed-dose combinations represents a combination of the drug abbreviation, dosage in milligram or gram, drug packaging, and the number of units per pack

### Trends in the available TB drugs

**Fig. 2:**
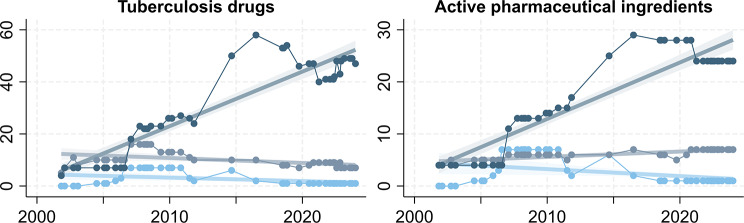
Tuberculosis drugs and active pharmaceutical ingredients listed in the Global Drug Facility Product Catalog, 2001–2024.  = single medicines,  = fixed-dose combinations,  = patient kits. P < 0.05 for all trends. N = 43

The number of TB drugs available from the GDF increased from 9 (8 APIs) in 2001 to 55 (32 APIs) in 2024. A peak was reached in 2016, when the GDF Product Catalog listed 70 drugs (38 APIs) and the largest selection of 58 single medicines (29 APIs) of all years. The number of FDCs peaked at 16 (6 APIs) in the years 2007 and 2008. The number of patient kits peaked at 7 between 2006 and 2010. Since 2018, only one patient kit has been listed in the product catalog. On average, the GDF Product Catalog contained 1.8 (95% CI: 1.4 to 2.1) more TB drugs per year. This increase was driven by the inclusion of 2.1 (95% CI: 1.9 to 2.3) additional single medicines per year and offset by reductions in the number of available FDC products (-0.2 [95% CI: -0.3 to -0.07] per year) and patient kits (-0.1 [95% CI: -0.2 to -0.03] per year) (Fig. 2 and supplementary Tables S4, S6).

As several TB drugs contain the same API(s) in different form, similar but less pronounced trends occurred for the number of APIs available from the GDF. The peak number of APIs listed in the GDF Product Catalogs was 29 (58 drugs) in 2016 for single medicines, 7 (7–10 drugs) in 2016 and from the end of 2020 to 2024 for FDCs, and 7 (7 drug regimens) from mid-2006 to 2010 for patient kits. The number of APIs available from the GDF increased on average by 1.1 (95% CI: 0.9 to 1.3) per year, resulting from increases in the APIs available as single medicines (0.1 [95% CI: 0.08 to 0.12] per year) as well as FDCs (0.1 [95% CI: 0.08 to 0.12] per year). The number of API combinations available as FDCs had an increasing trend despite a decreasing time trend in the number of FDC drugs (Fig. 2﻿﻿ and supplementary Tables S4, S6).

Specifying a stepwise change for the periods 2001–2006, 2007–2013, and 2014–2024 rather than a continuous time trend in the regression model explained more variation in the data. The average number of TB drugs listed in the GDF Product Catalog was, for instance, estimated at 17.3 (95% CI: 14.8 to 19.8) in the period 2001–2006, 43.8 (95% CI: 41.9 to 45.7) in the period 2007–2013, and 56.8 (95% CI: 54.5 to 59.1) in the period 2014–2024 (Wald-test *P* < 0.001) (supplementary Table S6).

### TB drug prices of the GDF

Drug product prices ranged from $0.66 to $1700 per pack of single medicines, from $1.33 to $99 per pack of FDCs, and from $11.56 to $98.90 per patient kit. The median (IQR) of the lowest prices from 2001 to 2024 was $13.38 (6.82–33) for single medicines, $21.96 (11.77–31.78) for FDCs, and $31.52 (21.50–54.21) for patient kits. The drug bedaquiline (Bdq) was listed at no purchasing cost in the 2016 product catalog due to a donation program available at the time. Prices including the bedaquiline donation price of zero were provided for 1651 (86.4%) of 1910 product listings. Price changes occurred in 646 (33.8%) of 1910 product listings. No prices were ever listed for 17 (9.8%) of 173 products in the GDF Product Catalog (Table 1 and supplementary Table S5).

### Trends in TB drug prices

#### Single medicines

**Fig. 3:**
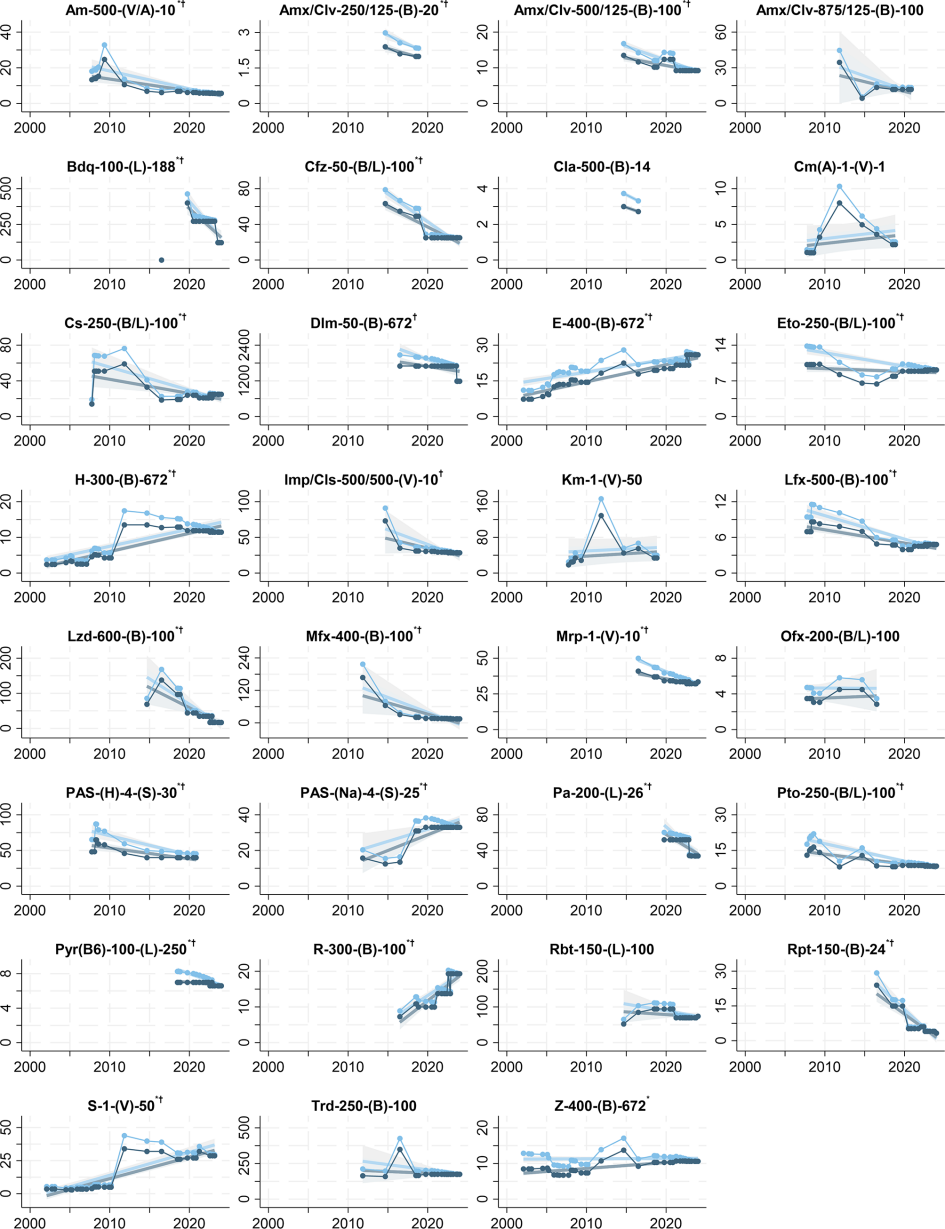
Prices of single medicines in the Global Drug Facility Product Catalog, 2001–2024.  = price in $. ^*^P < 0.05 for price trend.  = deflated price in $. ^†^P < 0.05 for deflated price trend. N = 9–40

We estimated significant price trends for 22 (71.0%) of the 31 APIs in the single medicines of the GDF Product Catalog. There was no unique price trend across single medicines. The price of 16 APIs decreased on average over time. The price of 6 APIs increased. The price of 9 APIs showed no trend. The five largest nominal price decreases were on average all larger than 10 pp per year (95% CI-bounds ranging from -26.35 to -4.99) and occurred for linezolid (Lzd), bedaquiline (Bdq), cycloserine (Cs), pretomanid (Pa), and rifapentine (Rpt). Average price increases were between 1.97 (95% CI: 1.58 to 2.36) to 45.15 (95% CI: 39.99 to 50.31) pp per year. The APIs with an increasing price trend were streptomycin (S), rifampicin (R), isoniazid (H), PAS sodium (PAS-[Na]), ethambutol (E), and pyrazinamide (Z). In monetary terms, the price trends of single medicines correspond to price decreases between -$0.08 (95% CI: -0.11 to -0.05) for Pyr(B6)-100-(L)-250 and -$50.43 (95% CI: -72.00 to -28.87) for Bdq-100-(L)-188 per year. Increasing price trend estimates were between $0.17 (95% CI: 0.13 to 0.20) for Z-400-(B)-672 and $1.76 (95% CI: 1.11 to 2.42) for PAS-(Na)-4-(S)-25 per year (Table 2, Fig. 3 and supplementary Fig. S2 blue plots).

**Table 2:** Time trends in the prices of active pharmaceutical ingredients and tuberculosis drugs in the Global Drug Facility Product Catalog, 2001–2024

**Product code with API**	**N**	API (pp per year)		TB drug ($ per year)	
		Normalized price	Deflated normalized price	Price	Deflated price
**Single medicines (31 APIs)**					
Am-500-(V/A)-10	28	-4.93 (-6.62 to -3.25)^*^	-5.53 (-7.17 to -3.89)^*^	-0.66 (-0.88 to -0.43)^*^	-1.00 (-1.30 to -0.71)^*^
Amx/Clv-250/125-(B)-20	5	-3.95 (-5.95 to -1.96)^*^	-5.07 (-6.96 to -3.19)^*^	-0.09 (-0.14 to -0.05)^*^	-0.15 (-0.21 to -0.10)^*^
Amx/Clv-500/125-(B)-100	21	-3.10 (-4.00 to -2.20)^*^	-4.63 (-5.42 to -3.84)^*^	-0.42 (-0.54 to -0.30)^*^	-0.78 (-0.91 to -0.65)^*^
Amx/Clv-875/125-(B)-100	9	-4.77 (-13.76 to 4.23)	-5.25 (-14.15 to 3.65)	-1.65 (-4.76 to 1.46)	-2.35 (-6.33 to 1.63)
Bdq-100-(L)-188	16	-12.61 (-18.00 to -7.22)^*^	-13.85 (-18.75 to -8.95)^*^	-50.43 (-72.00 to -28.87)^*^	-64.26 (-86.99 to -41.53)^*^
Cfz-50-(B/L)-100	22	-7.12 (-8.16 to -6.08)^*^	-7.88 (-8.85 to -6.91)^*^	-4.51 (-5.17 to -3.85)^*^	-6.22 (-6.98 to -5.45)^*^
Cla-500-(B)-14	2	-5.09	-6.08	-0.15	-0.23
Cm(A)-1-(V)-1	12	11.58 (-5.55 to 28.72)	8.82 (-7.40 to 25.04)	0.12 (-0.06 to 0.31)	0.13 (-0.11 to 0.36)
Cs-250-(B/L)-100	28	-11.20 (-17.40 to -4.99)^*^	-13.52 (-19.59 to -7.45)^*^	-1.58 (-2.46 to -0.71)^*^	-2.59 (-3.75 to -1.43)^*^
Dlm-50-(B)-672	20	-2.47 (-4.97 to 0.02)	-4.69 (-6.96 to -2.42)^*^	-42.05 (-84.45 to 0.36)	-97.35 (-144 to -50.25)^*^
E-400-(B)-672	40	9.85 (8.83 to 10.88)^*^	4.80 (3.90 to 5.71)^*^	0.72 (0.65 to 0.80)^*^	0.53 (0.43 to 0.64)^*^
Eto-250-(B/L)-100	28	-0.55 (-1.04 to -0.05)^*^	-1.90 (-2.30 to -1.50)^*^	-0.06 (-0.11 to -0.01)^*^	-0.26 (-0.32 to -0.21)^*^
H-300-(B)-672	40	20.95 (18.96 to 22.95)^*^	12.94 (10.99 to 14.89)^*^	0.51 (0.46 to 0.56)^*^	0.48 (0.41 to 0.55)^*^
Imp/Cls-500/500-(V)-10	21	-3.61 (-7.53 to 0.31)	-4.50 (-8.31 to -0.70)^*^	-2.64 (-5.51 to 0.23)	-4.10 (-7.57 to -0.64)^*^
Km-1-(V)-50	12	5.77 (-5.11 to 16.65)	3.44 (-6.87 to 13.74)	1.07 (-0.95 to 3.09)	0.87 (-1.73 to 3.46)
Lfx-500-(B)-100	28	-3.21 (-3.96 to -2.45)^*^	-4.17 (-4.88 to -3.46)^*^	-0.22 (-0.28 to -0.17)^*^	-0.39 (-0.46 to -0.33)^*^
Lzd-600-(B)-100	21	-16.60 (-26.35 to -6.85)^*^	-16.80 (-26.13 to -7.47)^*^	-11.45 (-18.18 to -4.73)^*^	-14.44 (-22.45 to -6.42)^*^
Mfx-400-(B)-100	22	-4.95 (-8.85 to -1.06)^*^	-5.09 (-9.01 to -1.16)^*^	-8.32 (-14.86 to -1.78)^*^	-11.04 (-19.56 to -2.53)^*^
Mrp-1-(V)-10	20	-2.42 (-3.14 to -1.71)^*^	-4.49 (-4.98 to -4.00)^*^	-0.99 (-1.29 to -0.70)^*^	-2.25 (-2.49 to -2.00)^*^
Ofx-200-(B/L)-100	9	1.07 (-5.20 to 7.34)	-0.06 (-5.81 to 5.68)	0.04 (-0.18 to 0.26)	-0.003 (-0.28 to 0.27)
PAS-(H)-4-(S)-30	15	-3.14 (-4.50 to -1.79)^*^	-4.09 (-5.41 to -2.77)^*^	-1.51 (-2.17 to -0.86)^*^	-2.67 (-3.54 to -1.81)^*^
PAS-(Na)-4-(S)-25	22	11.19 (7.07 to 15.32)^*^	6.69 (2.66 to 10.73)^*^	1.76 (1.11 to 2.42)^*^	1.36 (0.54 to 2.19)^*^
Pa-200-(L)-26	16	-10.47 (-15.06 to -5.89)^*^	-12.72 (-16.92 to -8.53)^*^	-5.45 (-7.83 to -3.06)^*^	-7.67 (-10.21 to -5.14)^*^
Pto-250-(B/L)-100	28	-3.19 (-4.02 to -2.37)^*^	-4.15 (-4.94 to -3.37)^*^	-0.42 (-0.52 to -0.31)^*^	-0.73 (-0.87 to -0.59)^*^
Pyr(B6)-100-(L)-250	19	-1.12 (-1.56 to -0.68)^*^	-3.95 (-4.56 to -3.33)^*^	-0.08 (-0.11 to -0.05)^*^	-0.33 (-0.38 to -0.28)^*^
R-300-(B)-100	20	23.70 (18.48 to 28.92)^*^	16.43 (12.66 to 20.19)^*^	1.73 (1.35 to 2.11)^*^	1.46 (1.13 to 1.80)^*^
Rbt-150-(L)-100	21	-2.84 (-10.43 to 4.75)	-5.75 (-13.57 to 2.07)	-1.49 (-5.48 to 2.49)	-3.76 (-8.87 to 1.35)
Rpt-150-(B)-24	20	-10.46 (-12.86 to -8.06)^*^	-10.71 (-13.22 to -8.21)^*^	-2.51 (-3.09 to -1.93)^*^	-3.14 (-3.87 to -2.40)^*^
S-1-(V)-50	31	45.15 (39.99 to 50.31)^*^	31.96 (27.14 to 36.78)^*^	1.63 (1.44 to 1.81)^*^	1.74 (1.48 to 2.01)^*^
Trd-250-(B)-100	22	-1.41 (-6.34 to 3.52)	-3.45 (-8.30 to 1.40)	-2.32 (-10.42 to 5.78)	-7.32 (-17.62 to 2.97)
Z-400-(B)-672	40	1.97 (1.58 to 2.36)^*^	0.08 (-0.30 to 0.46)	0.17 (0.13 to 0.20)^*^	0.01 (-0.04 to 0.06)
R^2^		0.028–0.924	0.0002–0.976	0.028–0.924	0.0002–0.976
**Fixed-dose combinations (9 API combinations)**					
3-HP-300/300-(B)-36	14	-10.49 (-17.35 to -3.62)^*^	-13.19 (-19.09 to -7.28)^*^	-1.57 (-2.60 to -0.54)^*^	-2.24 (-3.25 to -1.24)^*^
EH-400/150-(B)-672	21	16.42 (14.22 to 18.62)^*^	11.93 (9.87 to 13.99)^*^	1.30 (1.13 to 1.47)^*^	1.43 (1.18 to 1.68)^*^
HPST-Q-TIB-(L)-30	18	3.22 (1.31 to 5.14)^*^	-0.21 (-1.65 to 1.23)	0.06 (0.03 to 0.10)^*^	-0.005 (-0.04 to 0.03)
RH-150/150-(B)-672	20	22.52 (2.90 to 42.13)^*^	17.32 (0.20 to 34.43)^*^	1.81 (0.23 to 3.39)^*^	2.09 (0.02 to 4.16)^*^
RH-150/75-(B)-672	40	14.33 (12.98 to 15.67)^*^	7.82 (6.87 to 8.78)^*^	1.03 (0.93 to 1.13)^*^	0.85 (0.75 to 0.96)^*^
RH-75/50-(B)-84	22	37.94 (32.87 to 43.01)^*^	24.06 (22.25 to 25.87)^*^	0.19 (0.16 to 0.21)^*^	0.18 (0.17 to 0.19)^*^
RHE-150/75/275-(B)-672	37	9.60 (8.24 to 10.97)^*^	4.81 (3.72 to 5.91)^*^	1.53 (1.31 to 1.74)^*^	1.11 (0.86 to 1.36)^*^
RHZ-75/50/150-(B)-84	22	3.00 (-2.59 to 8.59)	1.07 (-3.94 to 6.07)	0.21 (-0.18 to 0.59)	0.09 (-0.35 to 0.54)
RHZE-150/75/400/275-(B)-672	40	8.70 (7.94 to 9.47)^*^	4.20 (3.62 to 4.78)^*^	1.78 (1.63 to 1.94)^*^	1.31 (1.12 to 1.49)^*^
R^2^		0.215–0.918	0.004–0.933	0.215–0.918	0.004–0.933
**Patient kits (8 API regimens)**					
PK-Cat I & III-A	37	7.22 (6.19 to 8.25)^*^	3.04 (2.14 to 3.94)^*^	0.83 (0.72 to 0.95)^*^	0.51 (0.36 to 0.66)^*^
PK-Cat I & III-B	12	4.61 (1.98 to 7.24)^*^	2.94 (0.41 to 5.46)^*^	1.04 (0.45 to 1.63)^*^	0.92 (0.13 to 1.71)^*^
PK-Cat I & III-C	12	1.00 (-0.09 to 2.09)	-0.34 (-1.33 to 0.64)	0.14 (-0.01 to 0.30)	-0.07 (-0.26 to 0.13)
PK-Cat II-A1	12	11.09 (7.78 to 14.39)^*^	8.47 (5.57 to 11.36)^*^	5.13 (3.60 to 6.66)^*^	5.48 (3.61 to 7.35)^*^
PK-Cat II-A2	12	6.68 (3.51 to 9.84)^*^	4.69 (1.89 to 7.49)^*^	2.80 (1.47 to 4.13)^*^	2.77 (1.12 to 4.43)^*^
PK-Cat II-B1	11	10.32 (7.68 to 12.95)^*^	7.98 (5.70 to 10.26)^*^	5.39 (4.01 to 6.77)^*^	5.80 (4.15 to 7.46)^*^
PK-Cat II-B2	10	8.03 (2.21 to 13.85)^*^	6.26 (0.88 to 11.63)^*^	3.46 (0.95 to 5.97)^*^	3.75 (0.53 to 6.98)^*^
PK-Cat II-C	1				
R^2^		0.184–0.853	0.031–0.807	0.184–0.853	0.031–0.807

API = active pharmaceutical ingredient. pp = percentage points. ( ) = 95% confidence interval. R^2^ for perfect fit is not reported. API prices were normalized such that the first listed price is 100. Price trend in $ estimated for the most listed TB drugs in the GDF Product Catalog using the lowest price per dose of the APIs

#### Fixed-dose combinations

**Fig. 4:**
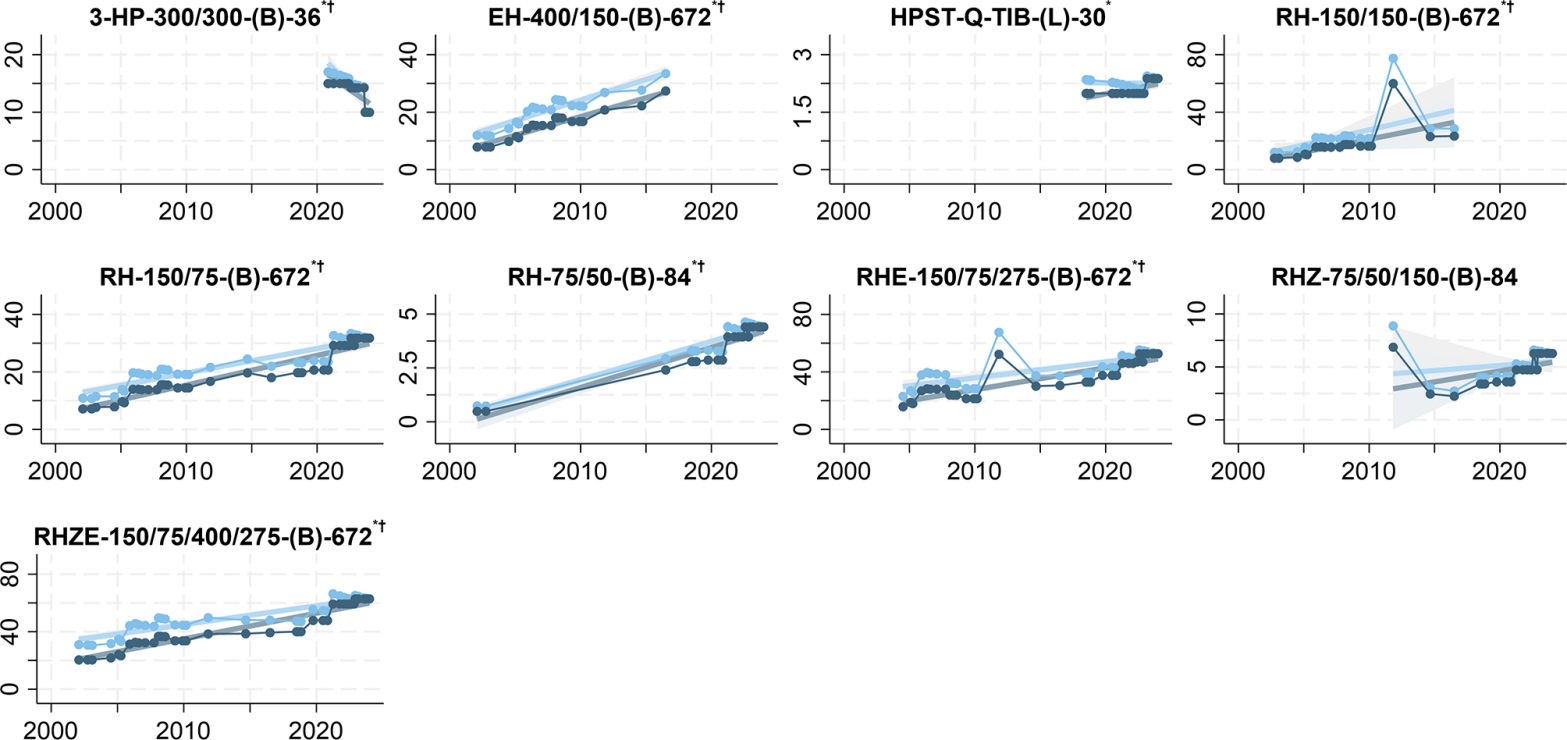
Prices of fixed-dose combinations in the Global Drug Facility Product Catalog, 2001–2024.  = price in $. ^*^P<0.05 for price trend.  = deflated price in $. ^†^P<0.05 for deflated price trend. N = 14–40

FDC tablets combined between 2 and 4 APIs, mostly consisting of rifampicin (R), isoniazid (H), pyrazinamide (Z), and/or ethambutol (E). FDCs of isoniazid, pyridoxine hydrochloride, sulfamethoxazole, and trimethoprim (HPST) and of rifapentine and isoniazid (3-HP) for TB preventive treatment became available in 2018 and 2020, respectively. We estimated price trends for 8 (88.9%) of the 9 API combinations used in FDCs. Only the price of 3-HP-300/300-(B)-36 had a decreasing trend (-10.49 [95% CI: -17.35 to -3.62] pp per year). Prices increased on average between 3.22 (95% CI: 1.31 to 5.14) and 37.94 (95% CI: 32.87 to 43.01) pp per year for HPST and most FDCs of EH, RH, RHZ, RHE. In monetary terms, the price of 3-HP-300/300-(B)-36 decreased on average by -$1.57 (95% CI: -2.60 to -0.54) per year. In monetary terms, the increasing price trends were between $0.06 (95% CI: 0.03 to 0.10) for HPST-Q-TIB-(L)-30 and $1.81 (95% CI: 0.23 to 3.39) for RH-150/150-(B)-672  per year and product (Table 2, Fig. 4 and supplementary Fig. S3 blue plots).

#### Patient kits

**Fig. 5:**
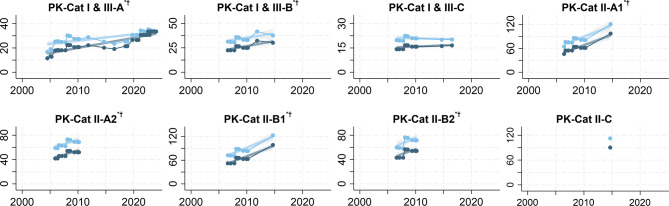
: Prices of patient kits in the Global Drug Facility Product Catalog, 2001–2024.  = price in $. ^*^P < 0.05 for price trend.  = deflated price in $. ^†^P < 0.05 for deflated price trend. N = 1–37

The price increased for 6 (75.0%) of 8 patient kits (PK-Cat I & III-A/B and PK-Cat II-A/B), showed no trend for 1 patient kit (PK-Cat I & III-C), and could not be estimated for another patient kit that was only listed once (PK-Cat II-C). The average price increase over the listing period ranged from 4.61 (95% CI: 1.98 to 7.24) pp per year for PK-Cat I & III-B to 11.09 (95% CI: 7.78 to 14.39) pp per year for PK-Cat II-A1. For PK-Cat I & III-A, which was available longest and the only patient kit in the 2024 GDF Product Catalog, the price increased on average by $0.83 (95% CI: 0.72 to 0.95) per year. This was the lowest significant price trend for patient kits. The steepest price trend was an additional $5.39 (95% CI: 4.01 to 6.77) per year for PK-Cat II-B1 (Table 2, Fig. 5 and supplementary Fig. S4 blue plots).

### Adjustment for price level changes

Adjusting GDF Product Catalog prices for gross domestic product deflation in advanced economies caused more pronounced price decreases and less pronounced price increases. Adjusting the price of single medicines resulted in estimating additional decreasing price trends for Dlm-50-(B)-672 and Imp/Cls-500/500-(V)-10. In addition, the deflated price of Z-400-(B)-672 ceased to have an increasing trend. Deflation adjustment of FDC prices eliminated the increasing price trend for HPST-Q-TIB-(L)-30. The significance of the price trends of patient kits was not affected by the price adjustment for deflation (Table 2, Fig. 3–5 and supplementary Fig. S2–S4 light blue plots).

## Discussion

### Summary of findings

This study described the availability and prices of single medicines, FDCs, and patient kits from the GDF, a major global supplier of TB drugs, and assessed time trends. The number of single medicines available from the GDF substantially increased between 2001 and 2024. Despite a decreasing number of FDCs and patient kits, more API combinations became available from the GDF as FDCs for TB preventive treatment were added to the product catalog. The lowest price of 16 APIs available as single medicines, 1 FDC, and no patient kit decreased over the period for which we had price information. An increasing price trend was estimated for 6 APIs available as single medicines, 7 API combinations available as FDCs, and 6 API regimens available as patient kits. Price adjustment resulted in estimating a decreasing price trend for 19 instead of 17 APIs and estimating an increasing price trend for 17 instead of 19 APIs. Price increases concentrated on the APIs of single medicines and FDCs used in the standard 6-month drug-susceptible TB treatment. The prices of several APIs used in new regimens against drug-resistant TB showed a decreasing trend. Price trends were mixed for APIs of single medicines and FDCs used in TB preventive treatment.

### Drug supply and prices by the Global Drug Facility

Availability of and access to quality-assured medicines are key components of TB control [2, 27]. The promotion of FDC tablets as a replacement for single medicines in TB control programs and their addition to national essential medicines lists were among the early priorities of the GDF [28]. In line with evolving needs and opportunities [29–31], the GDF Product Catalog expanded over time by listing several new drug products, including drugs in different dosage, packaging, and form. To purchase quality-assured TB drugs at competitive prices, the GDF pools demand and uses tendering among pre-qualified manufactures [7, 13]. In the past, prices for first-line and second-line TB drugs procured through the GDF were lower compared to the private market [14] or international tenders [7] and decreased in a short-term comparison for selected second-line TB drugs [32]. Our analysis showed that the GDF offered several APIs at prices that remained stable or decreased over the past two decades.

### Prices of drugs for BPaL and BPaLM regimens

The APIs with a decreasing price trend in our analysis include bedaquiline, linezolid, moxifloxacin, and pretomanid. All of them are used for drug-resistant TB treatment in some of the newest drug regimens. A short oral regimen of bedaquiline, pretomanid, and linezolid (BPaL) regimen was assessed in the Nix-TB study and included in the WHO guidelines for the treatment of drug-resistant TB in 2020 [33, 34]. The subsequent ZeNix trial found a BPaL regimen with a reduced linezolid (Lzd) dose of 600 mg instead of 1200 mg daily favorable [35]. The recently published TB-PRACTECAL trial favored a BPaLM (BPaL plus moxifloxacin) regimen over the local standard of care and also showed the high efficacy of the BPaL regimen with a reduced linezolid dose [36, 37]. The BPaLM and, alternatively, the BPaL regimen with a reduced dose of linezolid were included in WHO’s most recent 2022 guideline update [38, 39]. Given the positive treatment outcomes and substantially shortened treatment times, TB programs may increasingly need stable access to quality-assured bedaquiline, pretomanid, linezolid, and moxifloxacin.

The prices of bedaquiline, pretomanid, and linezolid decreased on average by 10.5 to 16.6 pp per year in our analysis. After the Janssen-USAID Bedaquiline Donation Program ended in early 2019 [23], the price of a pack 188 tablets bedaquiline (Bdq) 10 mg increased to $400 in the GDF Product Catalog. This price fell to $340 (plus a 20% free goods for each 10 packs ordered) in 2020. The price fell further to $244.40 (plus a 50% free goods for each 2 packs ordered) in 2023 and has been directly listed as $122.20 since the October 2023 GDF Product Catalog update. Twenty-six tablets pretomanid (Pa) 200 mg were mostly available for a price of $52 from 2019 to 2022. Their price decreased to $34.29 in the end of 2022 and to $33.95 since 2023. The price of dose equivalent of 100 tablets linezolid (Lzd) 600 mg had the strongest decreasing trend in the study. Their price decreased from $69.00 in 2014, after a jump to $137.90 in 2016, to $17.03 since the end of 2022. The price of 100 tablets moxifloxacin (Mfx) 400 mg decreased from $168 in 2011, to $64.51 in 2014, to a price between $15 and $16.90 since 2019. Considering the large cost contributions of pretomanid and bedaquiline to the price of BPaL and BPaLM regimens [15, 20], falling prices for these drugs contribute to substantial cost savings in TB treatment with BPaL and BPaLM regimens.

### Prices of drugs for drug-susceptible TB regimens

We found increasing price trends for all four APIs of the single medicines used in standard 6-month drug-susceptible TB treatment, namely for rifampicin (R), isoniazid (H), pyrazinamide (Z), and ethambutol (E). We also observed increasing price trends in the FDCs used in the intensive phase and continuation phase of standard drug-susceptible TB treatment. The price of the dose equivalent of 672 tablets RHZE in a 150 mg/75 mg/400 mg/275 mg ratio used during the intensive treatment phase increased from $20.50 in 2002 to $40.06 in 2018 and to $62.90 since the end of 2022. The price of the dose equivalent of 672 FDC tablets RH in a 150 mg/75 mg ratio used during the continuation phase increased from $7.19 in 2002 to $31.78 since the end of 2022. Although drug-susceptible TB drug regimen cost less than drug-resistant TB drug regimens [15], increasing prices for drugs used in drug-susceptible TB treatment could increase funding needs in TB care as drug-susceptible TB is more common than drug-resistant TB. The estimated 2022 global incidence of drug-resistant TB was 5.2 (95% uncertainty interval: 4.7 to 5.7) per 100,000 as compared to a total TB incidence of 133 (95% uncertainty interval: 124 to 143) per 100,000 [40].

The TBTC Study 31/A5349 showed that a 4-month regimen of rifapentine, moxifloxacin, isoniazid, and pyrazinamide was non-inferior to the standard 6-month drug regimen [41]. Since 2022, this new regimen has been included in the WHO recommendations for drug-susceptible TB treatment [39]. Contrary to the increasing price trends for most APIs used in the standard 6-month regimen against drug-susceptible TB, key APIs for the newer 4-month regimen have become available at lower prices from the GDF. A pack of 24 tablets rifapentine (Rpt) 150 mg was first listed in the GDF Product Catalog in 2016 with a price of $24 and has been available from the GDF at a price between $3.31 and $5.25 since 2020. Price reductions for rifapentine and moxifloxacin contribute to decreasing the costs of the 4-month drug-susceptible TB regimen.

### Prices of drugs for TB preventive treatment

For TB preventive treatment, WHO guidelines recommend a monotherapy of daily isoniazid for 6 or 9 months, a combination of weekly rifapentine and isoniazid for 3 months, a combination of daily isoniazid and rifampicin for 3 months, or a monotherapy of daily rifampicin for 4 months [42, 43]. Based on the BRIEF TB/A5279 Study [44], the 2020 WHO guideline update further recommended 1 month of daily rifapentine and isoniazid for TB preventive treatment [42]. In 2022, 1–3-month drug regimens were provided to 0.6 million out of 3.8 million people receiving TB preventive treatment, compared to 0.19 million out of 2.9 million people in 2021 [2]. The dose equivalent of 672 tablets isoniazid (H) 300 mg had an increasing trend from $2.45 in 2002 to a price between $11.48 and $13.52 since 2011. We also found trends for increasing prices for the dose-equivalent of 100 tablets of rifampicin (R) 300 mg, which could be obtained for $7.30 in 2016 and $19.33 since the end of 2022. Further, the prices of three different FDC tablets containing isoniazid and rifampicin and the price a FDC of isoniazid, pyridoxine hydrochloride, sulfamethoxazole, and trimethoprim (HPST), which is used for TB preventive treatment in adults living with HIV, increased. In turn, we found decreasing price trends for rifapentine and the FDC of isoniazid and rifapentine (HP). The price of 36 tablets 3-HP in a 300/300 ratio cost $15 in 2020 and $9.99 since the second half of 2023. As a result of these drug price trends, the prices of newer and older regimens are converging. This may contribute to the adoption of new rifapentine-based drug regimens in favor of other options for TB preventive treatment, which aligns with WHO’s recommendation to expand 1–3-month rifapentine/rifampicin-based drug regimens [2].

### Implication of trends in drug prices for TB programs

With $5.8 billion in 2022, funding for TB care in low- and middle-income countries reached 44% of its $13 billion target [2]. Considering this funding gap and the large contribution of TB drug prices to TB program costs [3, 4], drug price variations may have a profound impact on the number of people with TB that can receive treatment and on the TB drug regimens offered by TB programs. Decreasing prices for key drugs of new and shorter drug regimens for MDR-TB and drug-susceptible TB, such as bedaquiline, pretomanid, linezolid, moxifloxacin, and rifapentine, may facilitate the rollout of newer and shorter drug regimens. Increasing prices of drugs used in the 6-month drug-susceptible TB treatment may increase the funding needs of TB programs. In turn, a decreasing rifapentine price might facilitate the adoption of a rifapentine-based 4-month drug-susceptible TB regimen. To the extent that the presented findings are indicative of robust trends, this study can help TB programs optimize their choice of drug regimens considering treatment effectiveness, drug costs, and drug price trends.

### Strengths and limitations

This study is based on a comprehensive internet search through which past and recent GDF Product Catalogs were identified. Study limitations include, first, that the availability and prices of drug products were assessed based on product catalog entries, not GDF confirmed availability or actual purchases. Second, identified GDF Product Catalogs remain an incomplete subset of GDF drug products and prices. Third, drug prices that were only available through contacting the GDF were excluded from analysis. Fourth, price trends were assessed based on the lowest price per dose of an API. Reducing 158 TB drugs listed in GDF Product Catalogs to 48 APIs, API combinations, and API regimens facilitated the price comparison of drug products over time and provided a benchmark for the lowest price of an API. However, focusing on the lowest GDF prices of APIs assumed that drugs with the same API(s) can be arbitrarily multiplied or divided and substituted irrespective of their pharmaceutical form and packaging. This lowest price benchmark may not reflect the prices at which TB programs purchase a range of drugs with the same API(s) from the GDF. Fifth, we assessed a uniform linear trend over the longest period for which data was available even if some data plots suggested a low model fit.

Additional aspects to consider are that different drugs were available at different times and for different periods such that trend comparisons can have different reference periods. Inflation adjustment was based on the GDP deflator for advanced economics. While this might be a reasonable approximation for TB programs supported by donors, the purchasing power changes for specific countries could differ. Further, we analyzed prices at which TB programs could purchase TB drugs from the GDF, but these prices exclude additional import costs TB programs incur to provide drugs at the point of care [45]. Finally, we estimated trends in GDF prices without assessing underlying causes. How drug patents, production costs, new treatment guidelines, advocacy work, or the GDF as a major purchaser affect drug prices therefore remained unexplored in this study.

## Conclusions

TB programs benefit from a stable supply of low-priced quality-assured drugs and often procure through the GDF. We compiled GDF Product Catalogs since the GDF was launched in 2001 and assessed over 20 years of TB drug availability and price data. In line with growing pharmaceutical needs and opportunities in TB treatment, the number of single medicines listed in GDF Product Catalogs increased substantially over time. There was no unique price trend across the APIs available from the GDF in various drug forms. We found price increases for drugs used in standard 6-month drug-susceptible TB treatment and price reductions for drugs that are part of new drug-susceptible TB, drug-resistant TB, and TB preventive treatments, namely bedaquiline, pretomanid, linezolid, moxifloxacin, and rifapentine.

### Electronic supplementary material

Below is the link to the electronic supplementary material.

Supplementary Material 1

## Data Availability

The data and code that support the findings of this study are openly available in heiDATA at 10.11588/data/PIKWU6.
