# Supplementary material for: Trends in the availability and prices of quality-assured tuberculosis drugs: a systematic analysis of Global Drug Facility Product Catalogs from 2001 to 2024
Source: Global Health. 2024 Jun 25;20:51. doi: 10.1186/s12992-024-01047-7 (PMC11197363; doi:10.1186/s12992-024-01047-7)
Supplement: Supplementary file 1 — Supplementary Material 1 [file 12992_2024_1047_MOESM1_ESM.docx]

**Supplementary material**

**Table S1:** Abbreviations of single medicines

| **Product** | **Description** |
| --- | --- |
| Am | Amikacin |
| Amx/Clv | Amoxicillin/Clavulanic acid |
| Bdq | Bedaquiline |
| Cfz | Clofazimine |
| Cla | Clarithromycin |
| Cm | Capreomycin |
| Cs | Cycloserine |
| Dlm | Delamanid |
| E | Ethambutol |
| Eto | Ethionamide |
| H | Isoniazid |
| Imp/Cls | Imipenem/Cilastatin sodium |
| Km | Kanamycin |
| Lfx | Levofloxacin |
| Lzd | Linezolid |
| Mfx | Moxifloxacin |
| Mrp | Meropenem |
| Ofx | Ofloxacin |
| PAS-(H) | PAS acid |
| PAS-(Na) | PAS sodium |
| Pa | Pretomanid |
| Pto | Prothionamide |
| Pyr(B6) | Pyridoxine |
| R | Rifampicin |
| Rbt | Rifabutin |
| Rpt | Rifapentine |
| S | Streptomycin |
| Trd | Terizidone |
| Z | Pyrazinamide |

**Table S2:** Abbreviations of fixed-dose combinations

| **Product** | **Description** |
| --- | --- |
| 3-HP or 2-FDC/3-HP | Isoniazid/Rifapentine |
| EH or 2-FDC/EH | Ethambutol/Isoniazid |
| RH or 2-FDC/RH | Rifampicin/Isoniazid |
| RHE or 3-FDC/RHE | Rifampicin/Isoniazid/Ethambutol |
| RHZ or 3-FDC/RHZ | Rifampicin/Isoniazid/Pyrazinamide |
| HPST or 4-FDC/HPST-Q-TIB | Isoniazid/Pyridoxine hydrochloride/Sulfamethoxazole/Trimethoprim |
| RHZE or 4-FDC/RHZE | Rifampicin/Isoniazid/Pyrazinamide/Ethambutol |

#-FDC = fixed-dose combinations of # active pharmaceutical ingredients

**Table S3:** Abbreviations and description of patient kits

| **Product** | **Description** |
| --- | --- |
| PK-Cat I & III-A | Stop TB Cat. I+III Patient Kit A: 6 blisters (168) 4-FDC tablets (R150/H75/Z400/E275), 12 blisters (336) 2-FDC tablets (R150/H75) |
| PK-Cat I & III-B | Stop TB Cat. I+III Patient Kit B: 6 blisters (168) 4-FDC tablets (R150/H75/Z400/E275), 18 blisters (504) 2-FDC tablets (E400/H150) |
| PK-Cat I & III-C | Stop TB Cat. I+III Patient Kit C: 6 blisters (168) 4-FDC tablets (R150/H75/Z400/E275), 18 blisters (504) 2-FDC tablets (R150/H150) |
| PK-Cat II-A1 | Stop TB Cat. II Patient Kit A1: 9 blisters (252) 4-FDC tablets (R150/H75/Z400/E275), 56–60 vials of Streptomycin 1 gr, 60 vials of water for injection 5ml, 56–60 2-stroke auto-disabling syringes & needles, 15 blisters (420) 3-FDC tablets (R150/H75/E275) |
| PK-Cat II-A2 | Stop TB Cat. II Patient Kit A2: 9 blisters (252) 4-FDC tablets (R150/H75/Z400/E275), 56–60 vials of Streptomycin 1 gr, 60 vials of water for injection 5ml, 56–60 syringes & needles, 15 blisters (420) 3-FDC tablets (R150/H75/E275) |
| PK-Cat II-B1 | Stop TB Cat. II Patient Kit B1: 9 blisters (252) 4-FDC tablets (R150/H75/Z400/E275), 56–60 vials of Streptomycin 1 gr, 60 vials of water for injection 5ml, 56–60 2-stroke auto-disabling syringes & needles, 15 blisters (420) 2-FDC tablets (R150/H75), 10 blisters (280) tablets E400 |
| PK-Cat II-B2 | Stop TB Cat. II Patient Kit B2: 9 blisters (252) 4-FDC tablets (R150/H75/Z400/E275), 56–60 vials of Streptomycin 1 gr, 60 vials of water for injection 5ml, 56–60 syringes & needles, 15 blisters (420) 2-FDC tablets (R150/H75), 10 blisters (280) tablets E400 |
| PK-Cat II-C | Stop TB Cat. II Patient Kit C: 9 blisters (252) 4-FDC tablets (R150/H75/Z400/E275), 7 blisters (196) 2-FDC (RH 150/150), 7 blisters (196) E400, 56–60 vials of Streptomycin 1g, 60 vials of water for injection 5ml, 56–60 2-stroke auto-disabling syringes & needles, 1 Hub cutter |

Patient kits contain the full treatment course for one patient. PK-Cat I & III-A was initially named PK-Cat I & III. PK-Cat II-A1/A2 were initially named PK-Cat II. The number of streptomycin vials and syringes & needles varied between 56 and 60 for some patient kits across GDF Product Catalogs

**Table S4:** Number of TB drugs listed in the Global Drug Facility Product Catalog, 2001–2024

| **Year** | **Month** | **Single medicines** | | **Fixed-dose combinations** | | **Patient kits** | | **Total** | |
| --- | --- | --- | --- | --- | --- | --- | --- | --- | --- |
|  |  | **Drugs** | **APIs** | **Drugs** | **APIs** | **Drugs** | **APIs** | **Drugs** | **APIs** |
| 2001 | 11 | 4 | 4 | 5 | 4 | 0 | 0 | 9 | 8 |
| 2002 | 2 | 7 | 4 | 7 | 4 | 0 | 0 | 14 | 8 |
|  | 10 | 7 | 4 | 11 | 5 | 0 | 0 | 18 | 9 |
| 2003 | 2 | 7 | 4 | 8 | 4 | 0 | 0 | 15 | 8 |
| 2004 | 7 | 7 | 4 | 10 | 5 | 1 | 1 | 18 | 10 |
| 2005 | 2 | 7 | 4 | 10 | 5 | 1 | 1 | 18 | 10 |
|  | 4 | 7 | 4 | 10 | 5 | 1 | 1 | 18 | 10 |
|  | 12 | 7 | 4 | 10 | 5 | 2 | 2 | 19 | 11 |
| 2006 | 5 | 7 | 4 | 10 | 5 | 3 | 3 | 20 | 12 |
|  | 8 | 7 | 4 | 10 | 5 | 7 | 7 | 24 | 16 |
| 2007 | 2 | 18 | 11 | 16 | 6 | 7 | 7 | 41 | 24 |
|  | 10 | 23 | 13 | 16 | 6 | 7 | 7 | 46 | 26 |
| 2008 | 2 | 22 | 13 | 16 | 6 | 7 | 7 | 45 | 26 |
|  | 4 | 22 | 13 | 16 | 6 | 7 | 7 | 45 | 26 |
|  | 5 | 22 | 13 | 16 | 6 | 7 | 7 | 45 | 26 |
|  | 8 | 23 | 13 | 16 | 6 | 7 | 7 | 46 | 26 |
| 2009 | 5 | 23 | 13 | 13 | 6 | 7 | 7 | 43 | 26 |
|  | 12 | 26 | 14 | 13 | 6 | 7 | 7 | 46 | 27 |
| 2010 | 3 | 26 | 14 | 13 | 6 | 7 | 7 | 46 | 27 |
|  | 11 | 27 | 15 | 13 | 6 | 7 | 7 | 47 | 28 |
| 2011 | 7 | 26 | 15 | 11 | 5 | 3 | 3 | 40 | 23 |
|  | 11 | 24 | 17 | 10 | 6 | 2 | 2 | 36 | 25 |
| 2014 | 9 | 50 | 25 | 10 | 6 | 6 | 6 | 66 | 37 |
| 2016 | 7 | 58 | 29 | 10 | 7 | 2 | 2 | 70 | 38 |
| 2018 | 7 | 53 | 28 | 8 | 6 | 1 | 1 | 62 | 35 |
|  | 8 | 53 | 28 | 8 | 6 | 1 | 1 | 62 | 35 |
|  | 11 | 54 | 28 | 8 | 6 | 1 | 1 | 63 | 35 |
| 2019 | 10 | 46 | 28 | 7 | 5 | 1 | 1 | 54 | 34 |
| 2020 | 7 | 47 | 28 | 8 | 6 | 1 | 1 | 56 | 35 |
|  | 11 | 47 | 28 | 9 | 7 | 1 | 1 | 57 | 36 |
| 2021 | 4 | 40 | 24 | 9 | 7 | 1 | 1 | 50 | 32 |
|  | 10 | 41 | 24 | 9 | 7 | 1 | 1 | 51 | 32 |
| 2022 | 2 | 41 | 24 | 9 | 7 | 1 | 1 | 51 | 32 |
|  | 5 | 41 | 24 | 9 | 7 | 1 | 1 | 51 | 32 |
|  | 6 | 42 | 24 | 9 | 7 | 1 | 1 | 52 | 32 |
|  | 8 | 48 | 24 | 7 | 7 | 1 | 1 | 56 | 32 |
|  | 11 | 43 | 24 | 9 | 7 | 1 | 1 | 53 | 32 |
|  | 12 | 48 | 24 | 7 | 7 | 1 | 1 | 56 | 32 |
| 2023 | 3 | 49 | 24 | 7 | 7 | 1 | 1 | 57 | 32 |
|  | 8 | 49 | 24 | 7 | 7 | 1 | 1 | 57 | 32 |
|  | 9 | 49 | 24 | 7 | 7 | 1 | 1 | 57 | 32 |
|  | 10 | 49 | 24 | 7 | 7 | 1 | 1 | 57 | 32 |
| 2024 | 1 | 47 | 24 | 7 | 7 | 1 | 1 | 55 | 32 |
| 2001–2024 | | 122 | 31 | 28 | 9 | 8 | 8 | 158 | 48 |
| Mean (SD) | | 31.3 (17.4) | 17.2 (9.02) | 10 (3.04) | 6 (0.926) | 3 (2.7) | 3 (2.7) | 44 (16.5) | 25.9 (9.5) |
| Median (IQR) | | 27 (18–48) | 17 (11–24) | 9 (8–11) | 6 (5–7) | 1 (1–7) | 1 (1–7) | 47 (36–56) | 28 (23–32) |
| Range | | 4–58 | 4–29 | 5–16 | 4–7 | 1–7 | 1–7 | 9–70 | 8–38 |

API = active pharmaceutical ingredient, IQR = interquartile range, SD = standard deviation

**Table S5:** TB drugs, formulations, and prices in the Global Drug Facility Product Catalog, 2001–2024

| **Product code** | **Dose** | **Mean price (SD)** | **Median price (IQR)** | **Price per dose** | | | **API** |
| --- | --- | --- | --- | --- | --- | --- | --- |
|  |  |  |  | **Lowest price (¢)** | **Minimum range (pp)** | **Maximum range (pp)** |  |
| **Single medicines (N = 122)** | | | | | | | |
| Am-500-(A)-1 | 500 | 1.00 | 1.00 | 0.200 | 100–247 | 100–398 | Am |
| Am-500-(V)-5 | 500 | 8.25 | 8.25 | 0.330 |  |  |  |
| Am-500-(V)-20 | 500 | 49.47 | 49.47 | 0.495 |  |  |  |
| Am-500-(V/A)-10 | 500 | 8.68 (0.77) | 6.71 (6.51–12.00) | 0.124 |  |  |  |
| Am-500-(V/A)-100 | 500 | 58.19 (0.34) | 57.98 (57.20–58.75) | 0.114 |  |  |  |
| Amx/Clv-250/125-(B)-14 | 375 | 1.82 (0.19) | 1.63 (1.63–2.00) | 0.031 | 100–136 | 100–210 | Amx/Clv-2/1 |
| Amx/Clv-250/125-(B)-20 | 375 | 2.09 (0.08) | 1.99 (1.99–2.10) | 0.027 |  |  |  |
| Amx/Clv-125/31.25-(BTL)-1 | 156.25 | 1.31 (0.10) | 1.21 (1.21–1.42) | 0.774 | 100–208 | 100–7654 | Amx/Clv-4/1 |
| Amx/Clv-250/62.5-(BTL)-1 | 312.5 | 1.94 (0.08) | 1.94 (1.86–2.03) | 0.595 |  |  |  |
| Amx/Clv-500/125-(B)-15 | 625 | 1.84 (0.09) | 1.75 (1.75–1.75) | 0.019 |  |  |  |
| Amx/Clv-500/125-(B)-20 | 625 | 2.70 | 2.70 | 0.022 |  |  |  |
| Amx/Clv-500/125-(B)-21 | 625 | 2.63 | 2.63 | 0.020 |  |  |  |
| Amx/Clv-500/125-(B)-100 | 625 | 10.34 (0.40) | 9.25 (9.25–10.21) | 0.015 |  |  |  |
| Amx/Clv-875/125-(B)-12 | 1000 | 1.73 (0.04) | 1.69 (1.69–1.69) | 0.014 | 100–519 | 100–785 | Amx/Clv-7/1 |
| Amx/Clv-875/125-(B)-14 | 1000 | 4.84 | 4.84 | 0.035 |  |  |  |
| Amx/Clv-875/125-(B)-16 | 1000 | 2.16 | 2.16 | 0.013 |  |  |  |
| Amx/Clv-875/125-(B)-100 | 1000 | 12.26 (1.77) | 11.80 (11.80–11.80) | 0.004 |  |  |  |
| Bdq-20-(L)-60 | 20 | 25.53 | 25.53 (25.53–25.53) | 2.13 | 100–327 | 100–327 | Bdq |
| Bdq-100-(B)-100 | 100 | 92.60 (4.37) | 96.97 (88.23–96.97) | 0.795 |  |  |  |
| Bdq-100-(L)-188 | 100 | 243 (19.61) | 272 (197–272) | 0.650 |  |  |  |
| Cfz-50-(B/L)-100 | 50 | 42.94 (1.42) | 40.40 (35.78–49.12) | 0.716 | 100–181 | 100–181 | Cfz |
| Cfz-100-(B/L)-100 | 100 | 63.38 (5.49) | 50.00 (50.00–50.00) | 0.500 |  |  |  |
| Cla-250-(B)-14 | 250 | 1.75 (0.15) | 1.75 (1.60–1.90) | 0.046 | 100–229 | 100–295 | Cla |
| Cla-500-(B)-14 | 500 | 2.86 (0.14) | 2.86 (2.72–3.00) | 0.039 |  |  |  |
| Cm-0.5-(V)-1 | 0.5 | 2.99 | 2.99 | 598 | 100–100 | 100–249 | Cm |
| Cm-0.5-(V)-80 | 0.5 | 239 | 239 | 598 |  |  |  |
| Cm-0.75-(V)-1 | 0.75 | 3.99 | 3.99 | 532 |  |  |  |
| Cm-0.75-(V)-80 | 0.75 | 319 | 319 | 532 |  |  |  |
| Cm(A)-1-(V)-1 | 1 | 3.21 | 3.21 (3.21–3.21) | 321 |  |  |  |
| Cm(B)-1-(V)-1 | 1 | 1.03 (0.01) | 1.02 (1.02–1.02) | 102 |  |  |  |
| Cm-1-(V)-10 | 1 | 21.70 | 21.70 (21.70–21.70) | 217 |  |  |  |
| Cm-1-(V/A)-1 | 1 | 4.41 (0.76) | 3.46 (3.31–4.95) | 331 |  |  |  |
| Cs-125-(B)-100 | 125 | 44.61 (0.21) | 45.00 (45.00–45.00) | 0.340 | 100–406 | 100–468 | Cs |
| Cs-250-(B)-21 | 250 | 7.43 | 7.43 | 0.142 |  |  |  |
| Cs-250-(B)-40 | 250 | 7.43 | 7.43 | 0.074 |  |  |  |
| Cs(A)-250-(B)-100 | 250 | 50.96 | 50.96 | 0.204 |  |  |  |
| Cs(B)-250-(B)-100 | 250 | 14.12 | 14.12 | 0.056 |  |  |  |
| Cs-250-(B/L)-100 | 250 | 31.88 (2.84) | 25.00 (21.00–42.00) | 0.077 |  |  |  |
| Dlm-25-(B-DT)-48 | 25 | 85.00 | 85.00 (85.00–85.00) | 7.08 | 100–100 | 100–140 | Dlm |
| Dlm-50-(B)-48 | 50 | 94.11 (9.11) | 85.00 (85.00–103) | 3.54 |  |  |  |
| Dlm-50-(B)-672 | 50 | 1700 | 1700 (1700–1700) | 5.06 |  |  |  |
| E-100-(B)-500 | 100 |  |  |  | 100–322 | 100–7751 | E |
| E-100-(B-DT)-100 | 100 | 22.19 (0.49) | 21.25 (20.05–25.00) | 0.201 |  |  |  |
| E-100-(B/L)-100 | 100 | 3.68 (0.05) | 3.69 (3.58–3.95) | 0.030 |  |  |  |
| E-400-(B)-672 | 400 | 18.72 (0.78) | 19.50 (14.90–21.65) | 0.003 |  |  |  |
| E-400-(L)-1000 | 400 | 18.23 (1.08) | 20.17 (13.72–21.48) | 0.003 |  |  |  |
| E-600-(B)-100 | 600 | 4.00 | 4.00 | 0.007 |  |  |  |
| E-800-(B)-100 | 800 | 11.11 | 11.11 | 0.014 |  |  |  |
| Eto-125-(B)-90 | 125 | 10.06 | 10.06 | 0.089 | 100–173 | 100–664 | Eto |
| Eto-125-(B-DT)-100 | 125 | 14.36 (0.67) | 13.30 (13.00–13.30) | 0.104 |  |  |  |
| Eto-125-(B/L)-100 | 125 | 8.61 (0.46) | 8.14 (8.14–9.07) | 0.065 |  |  |  |
| Eto-250-(B)-10 | 250 |  |  |  |  |  |  |
| Eto-250-(B)-90 | 250 | 5.94 | 5.94 | 0.026 |  |  |  |
| Eto-250-(B/L)-100 | 250 | 9.01 (0.19) | 8.92 (8.92–9.16) | 0.026 |  |  |  |
| H-100-(B-DT)-100 | 100 | 9.43 (0.13) | 9.91 (8.95–9.91) | 0.085 | 100–360 | 100–5362 | H |
| H-100-(B/L)-100 | 100 | 1.29 (0.09) | 1.24 (1.09–1.29) | 0.008 |  |  |  |
| H-100-(L)-1000 | 100 |  |  |  |  |  |  |
| H-300-(B)-672 | 300 | 9.14 (0.55) | 11.48 (6.04–11.86) | 0.002 |  |  |  |
| H-300-(L)-1000 | 300 | 5.38 (0.39) | 4.98 (3.76–6.83) | 0.001 |  |  |  |
| Imp/Cls-500/500-(V)-1 | 1000 | 7.32 | 7.32 | 0.732 | 100–126 | 100–373 | Imp/Cls-1/1 |
| Imp/Cls-500/500-(V)-10 | 1000 | 29.77 (0.35) | 29.40 (28.50–30.50) | 0.285 |  |  |  |
| Km-0.5-(V)-1 | 0.5 |  |  |  | 100–605 | 100–694 | Km |
| Km-0.5-(V)-10 | 0.5 | 5.50 | 5.50 | 110 |  |  |  |
| Km-0.5-(V)-50 | 0.5 | 42.50 | 42.50 (42.50–42.50) | 170 |  |  |  |
| Km-1-(V)-1 | 1 |  |  |  |  |  |  |
| Km-1-(V)-50 | 1 | 37.82 (7.76) | 33.96 (24.88–34.00) | 37.16 |  |  |  |
| Km-1-(V/A)-10 | 1 | 24.21 (0.50) | 23.48 (23.36–25.80) | 234 |  |  |  |
| Lfx-100-(B-DT)-100 | 100 | 23.75 (6.25) | 12.41 (11.86–12.91) | 0.119 | 100–409 | 100–11364 | Lfx |
| Lfx-250-(B)-100 | 250 | 3.21 (0.19) | 2.65 (2.63–3.92) | 0.010 |  |  |  |
| Lfx-500-(B)-80 | 500 | 5.62 | 5.62 | 0.014 |  |  |  |
| Lfx-500-(B)-100 | 500 | 5.65 (0.36) | 4.82 (4.53–6.98) | 0.008 |  |  |  |
| Lfx-750-(B)-100 | 750 | 9.82 (0.06) | 9.90 (9.52–9.90) | 0.013 |  |  |  |
| Lzd-150-(B-DT)-100 | 150 | 27.27 | 27.27 (27.27–27.27) | 0.182 | 100–822 | 100–893 | Lzd |
| Lzd-600-(B)-10 | 600 | 13.49 (0.10) | 13.39 (13.39–13.59) | 0.223 |  |  |  |
| Lzd-600-(B)-100 | 600 | 39.59 (6.32) | 34.90 (17.03–44.20) | 0.028 |  |  |  |
| Lzd-600-(B)-200 | 600 | 138 | 138 | 0.115 |  |  |  |
| Mfx-100-(B-DT)-100 | 100 | 29.88 (5.51) | 19.90 (19.60–19.90) | 0.196 | 100–430 | 100–2267 | Mfx |
| Mfx-400-(B)-5 | 400 | 4.54 (1.97) | 3.30 (1.93–8.40) | 0.096 |  |  |  |
| Mfx-400-(B)-100 | 400 | 19.50 (2.39) | 16.00 (15.00–16.90) | 0.037 |  |  |  |
| Mrp-1-(V)-1 | 1 | 3.80 (0.10) | 3.70 (3.70–3.90) | 370 | 100–108 | 100–127 | Mrp |
| Mrp-1-(V)-10 | 1 | 33.18 (0.21) | 33.57 (32.20–33.57) | 322 |  |  |  |
| Ofx-200-(B/L)-100 | 200 | 3.84 (0.19) | 3.49 (3.49–4.02) | 0.017 | 100–169 | 100–169 | Ofx |
| Ofx-400-(B/L)-100 | 400 | 7.12 (0.61) | 6.44 (6.14–9.00) | 0.014 |  |  |  |
| PAS-(H)-4-(S)-30 | 4 | 47.30 (2.49) | 40.00 (40.00–57.98) | 33.33 | 100–162 | 100–162 | PAS-(H) |
| PAS-(Na)-4-(S)-25 | 4 | 33.04 (0.04) | 33.00 (33.00–33.00) | 33.00 | 100–102 | 100–102 | PAS-(Na) |
| PAS-(Na)-4-(S)-30 | 4 | 37.20 | 37.20 (37.20–37.20) | 31.00 |  |  |  |
| PAS-(Na)-5.52-(S)-25 | 5.52 | 35.88 (1.62) | 35.88 (34.25–37.50) | 24.82 |  |  |  |
| PAS-(Na)-9.2-(S)-30 | 9.2 | 39.00 (4.50) | 39.00 (34.50–43.50) | 12.50 |  |  |  |
| PAS-(Na)-100-(L)-1 | 100 | 14.00 (1.04) | 13.50 (12.49–16.00) | 12.49 |  |  |  |
| Pa-200-(B)-100 | 200 | 131 | 131 (131–131) | 0.653 | 100–152 | 100–152 | Pa |
| Pa-200-(L)-26 | 200 | 45.36 (2.21) | 52.00 (34.29–52.00) | 0.659 |  |  |  |
| Pto-250-(B)-140 | 250 | 18.10 | 18.10 | 0.052 | 100–201 | 100–201 | Pto |
| Pto-250-(B/L)-100 | 250 | 10.36 (0.56) | 8.74 (8.43–13.58) | 0.033 |  |  |  |
| Pto-250-(L)-50 | 250 | 4.07 | 4.07 | 0.033 |  |  |  |
| Pyr(B6)-10-(B)-100 | 10 | 3.00 | 3.00 (3.00–3.00) | 0.300 | 100–140 | 100–909 | Pyr(B6) |
| Pyr(B6)-50-(B)-50 | 50 | 0.69 | 0.70 (0.66–0.70) | 0.026 |  |  |  |
| Pyr(B6)-100-(L)-250 | 100 | 10.73 (0.27) | 10.75 (10.75–11.55) | 0.033 |  |  |  |
| R-150-(B)-80 | 150 |  |  |  | 100–342 | 100–407 | R |
| R-150-(B)-100 | 150 | 9.09 (0.71) | 8.80 (6.33–12.85) | 0.025 |  |  |  |
| R-150-(B)-800 | 150 |  |  |  |  |  |  |
| R-300-(B)-40 | 300 |  |  |  |  |  |  |
| R-300-(B)-100 | 300 | 15.18 (0.94) | 13.77 (11.88–19.33) | 0.024 |  |  |  |
| Rbt-150-(B)-24 | 150 | 12.60 | 12.60 | 0.350 | 100–135 | 100–179 | Rbt |
| Rbt-150-(L)-100 | 150 | 78.22 (2.56) | 69.86 (69.86–94.43) | 0.466 |  |  |  |
| Rpt-150-(B)-24 | 150 | 8.61 (1.16) | 6.00 (6.00–11.07) | 0.146 | 100–457 | 100–457 | Rpt |
| Rpt-150-(B-DT)-100 | 150 | 13.80 | 13.80 | 0.092 |  |  |  |
| Rpt-300-(B)-100 | 300 | 33.92 | 33.92 (33.92–33.92) | 0.113 |  |  |  |
| S-0.75-(V)-50 | 0.75 | 2.70 | 2.70 (2.70–2.70) | 7.20 | 100–177 | 100–1153 | S |
| S-1-(V)-10 | 1 | 5.46 (0.08) | 5.41 (5.22–5.75) | 52.20 |  |  |  |
| S-1-(V)-50 | 1 | 4.20 (0.24) | 3.89 (3.51–5.19) | 5.90 |  |  |  |
| S-1-(V)-100 | 1 | 64.57 (0.57) | 64.00 (64.00–64.00) | 64.00 |  |  |  |
| Trd-250-(B)-50 | 250 | 97.66 (15.38) | 83.30 (82.13–83.30) | 0.635 | 100–100 | 100–199 | Trd |
| Trd-250-(B)-100 | 250 | 175 | 175 (175–175) | 0.700 |  |  |  |
| Z-150-(B-DT)-100 | 150 | 11.76 (1.15) | 14.50 (9.40–14.94) | 0.009 | 100–182 | 100–3283 | Z |
| Z-150-(B/L)-100 | 150 |  |  |  |  |  |  |
| Z-150-(L)-1000 | 150 |  |  |  |  |  |  |
| Z-400-(B)-672 | 400 | 11.88 (0.35) | 13.38 (9.81–14.00) | 0.003 |  |  |  |
| Z-400-(L)-1000 | 400 | 11.52 (0.26) | 11.98 (10.14–12.64) | 0.003 |  |  |  |
| Z-500-(B)-100 | 500 | 2.14 (0.42) | 2.14 (1.72–2.56) | 0.003 |  |  |  |
| Z-500-(B)-120 | 500 | 6.25 | 6.25 (6.25–6.25) | 0.010 |  |  |  |
| Z-500-(B)-672 | 500 | 13.95 (0.51) | 13.31 (12.91–13.40) | 0.004 |  |  |  |
| Z-750-(B)-672 | 750 | 31.00 | 31.00 | 0.006 |  |  |  |
| **Fixed-dose combinations (N = 28)** | | | | | | | |
| 3-HP-300/300-(B)-36 | 600 | 13.66 (0.54) | 14.25 (14.25–15.00) | 0.046 | 100–150 | 100–150 | 3-HP |
| EH-400/150-(B)-672 | 550 | 17.48 (1.02) | 17.24 (15.39–20.72) | 0.002 | 100–379 | 100–379 | EH |
| EH-400/150-(L)-1000 | 550 | 21.09 (1.32) | 22.92 (16.58–25.00) | 0.002 |  |  |  |
| HPST-Q-TIB-(L)-30 | 1285 | 2.10 (0.04) | 1.99 (1.99–2.38) | 0.005 | 100–120 | 100–120 | HPST-Q-TIB |
| RH-75/50-(B)-84 | 125 | 3.70 (0.16) | 3.95 (2.87–4.41) | 0.023 | 100–197 | 100–197 | RH |
| RH-150/100-(B)-672 | 250 | 8.87 | 8.87 (8.87–8.87) | 0.005 |  |  |  |
| RH-150/100-(L)-1000 | 250 | 11.66 | 11.66 (11.66–11.66) | 0.005 |  |  |  |
| RH-60/60-(B)-80 | 120 |  |  |  | 100–252 | 100–645 | RH-1/1 |
| RH-60/60-(B)-84 | 120 | 3 | 3.00 (3.00–3.00) | 0.03 |  |  |  |
| RH-60/60-(L)-1000 | 120 |  |  |  |  |  |  |
| RH-150/150-(B)-672 | 300 | 17.92 (1.04) | 19.22 (12.74–21.07) | 0.005 |  |  |  |
| RH-150/150-(L)-1000 | 300 | 21.41 (1.29) | 23.64 (16.22–24.45) | 0.004 |  |  |  |
| RH-60/30-(B)-84 | 90 | 2.27 (0.87) | 1.47 (1.33–4.00) | 0.018 | 100–399 | 100–909 | RH-2/1 |
| RH-60/30-(B)-90 | 90 |  |  |  |  |  |  |
| RH-60/30-(L)-1000 | 90 |  |  |  |  |  |  |
| RH-150/75-(B)-336 | 225 | 14.13 (0.51) | 14.64 (12.60–15.65) | 0.015 |  |  |  |
| RH-150/75-(B)-672 | 225 | 21.76 (1.15) | 19.78 (17.97–29.23) | 0.006 |  |  |  |
| RH-150/75-(L)-1000 | 225 | 19.38 (1.07) | 20.86 (14.45–23.07) | 0.005 |  |  |  |
| RH-300/150-(L)-1000 | 450 | 21.4 | 21.40 (21.40–21.40) | 0.005 |  |  |  |
| RHE-150/75/275-(B)-672 | 500 | 36.94 (1.88) | 33.00 (27.02–46.99) | 0.005 | 100–556 | 100–556 | RHE |
| RHE-150/75/275-(L)-1000 | 500 | 38.09 (2.89) | 35.72 (31.99–41.80) | 0.005 |  |  |  |
| RHZ-60/30/150-(B)-84 | 240 | 3.37 (1.32) | 2.14 (1.96–6.00) | 0.010 | 100–225 | 100–233 | RHZ |
| RHZ-60/30/150-(B)-90 | 240 |  |  |  |  |  |  |
| RHZ-60/30/150-(L)-1000 | 240 |  |  |  |  |  |  |
| RHZ-75/50/150-(B)-84 | 275 | 4.82 (0.28) | 4.74 (3.60–6.28) | 0.013 |  |  |  |
| RHZE-150/75/400/275-(B)-336 | 900 | 28.22 (0.90) | 29.11 (25.52–30.93) | 0.008 | 100–313 | 100–313 | RHZE |
| RHZE-150/75/400/275-(B)-672 | 900 | 44.27 (2.12) | 40.06 (36.11–59.33) | 0.004 |  |  |  |
| RHZE-150/75/400/275-(L)-1000 | 900 | 45.93 (2.15) | 48.36 (35.40–54.57) | 0.003 |  |  |  |
| **Patient kits (N = 8)** | | | | | | | |
| **Product code** | **Dose** | **Mean price (SD)** | **Median price (IQR)** | **Lowest price ($)** | **Minimum range (pp)** | **Maximum range (pp)** | **API** |
| PK-Cat I & III-A | 1 | 24.47 (1.09) | 22.40 (20.23–30.77) | 11.56 | 100–304 | 100–304 | I & III-A |
| PK-Cat I & III-B | 1 | 25.98 (0.86) | 25.81 (23.80–26.67) | 22.54 | 100–143 | 100–143 | I & III-B |
| PK-Cat I & III-C | 1 | 15.82 (0.29) | 16.01 (15.05–16.66) | 14.26 | 100–117 | 100–117 | I & III-C |
| PK-Cat II-A1 | 1 | 62.38 (3.55) | 61.50 (54.94–63.91) | 46.27 | 100–211 | 100–211 | II-A1 |
| PK-Cat II-A2 | 1 | 49.52 (1.42) | 52.08 (45.80–54.18) | 41.90 | 100–129 | 100–129 | II-A2 |
| PK-Cat II-B1 | 1 | 64.52 (3.87) | 63.33 (52.27–66.05) | 52.27 | 100–189 | 100–189 | II-B1 |
| PK-Cat II-B2 | 1 | 51.85 (1.93) | 54.21 (43.13–56.59) | 43.13 | 100–131 | 100–131 | II-B2 |
| PK-Cat II-C | 1 | 90.40 | 90.40 | 90.40 | 100–100 | 100–100 | II-C |

API = active pharmaceutical ingredient, IQR = interquartile range, SD = standard deviation, pp = percentage points. DS = drug-susceptible, DR = drug-resistant, TPT = TB preventive treatment. (A) = ampoule, (BTL) = bottle, (B) = blister, (B-DT) = blistered dispersible tablets, (B/L) = blister or loose (L) = loose, (S) = sachet, (V) = vial, (V/A) = vial or ampoule. The product code of single medicines and fixed-dose combinations represents a combination of the drug abbreviation, dosage in milligram or gram, drug packaging, and the number of units per pack

**Table S6:** Trends in the number of tuberculosis drugs listed in the Global Drug Facility Product Catalog, 2001–2024

|  | **Single medicines** | **Fixed-dose combinations** | **Patient kits** | **All drug products** |
| --- | --- | --- | --- | --- |
| **Number of drugs** | | | | |
| ***Model 1*** | | | | |
| Time (years) | 2.1 (1.9 to 2.3)^*^ | -0.2 (-0.3 to -0.07)^*^ | -0.1 (-0.2 to -0.03)^*^ | 1.8 (1.4 to 2.1)^*^ |
| Constant | -4189 (-4608 to -3770)^*^ | 394 (144 to 644)^*^ | 272 (54.1 to 490)^*^ | -3523 (-4221 to -2825)^*^ |
| R^2^ | 0.847 | 0.231 | 0.142 | 0.678 |
| R^2^-adjusted | 0.843 | 0.212 | 0.122 | 0.670 |
| ***Model 2*** | | | | |
| 2007–2013 | 16.8 (15.2 to 18.4)^*^ | 5.0 (3.3 to 6.7)^*^ | 4.7 (3.0 to 6.5)^*^ | 26.5 (23.4 to 29.7)^*^ |
| 2014–2023 | 40.7 (38.4 to 42.9)^*^ | -1.0 (-2.2 to 0.3) | -0.2 (-1.7 to 1.2) | 39.5 (36.1 to 42.9)^*^ |
| Constant | 6.7 (6.1 to 7.3)^*^ | 9.1 (7.9 to 10.3)^*^ | 1.5 (0.1 to 2.9)^*^ | 17.3 (14.8 to 19.8)^*^ |
| R^2^ | 0.958 | 0.725 | 0.673 | 0.929 |
| R^2^-adjusted | 0.955 | 0.711 | 0.656 | 0.925 |
| ***Model 3*** | | | | |
| 2001–2006 | 6.7 (6.1 to 7.3)^*^ | 9.1 (7.9 to 10.3)^*^ | 1.5 (0.1 to 2.9)^*^ | 17.3 (14.8 to 19.8)^*^ |
| 2007–2013 | 23.5 (22.0 to 25.0)^*^ | 14.1 (12.8 to 15.4)^*^ | 6.3 (5.2 to 7.3)^*^ | 43.8 (41.9 to 45.7)^*^ |
| 2014–2023 | 47.4 (45.2 to 49.5)^*^ | 8.1 (7.7 to 8.6)^*^ | 1.3 (0.8 to 1.8)^*^ | 56.8 (54.5 to 59.1)^*^ |
| R^2^ | 0.990 | 0.977 | 0.839 | 0.991 |
| R^2^-adjusted | 0.989 | 0.976 | 0.827 | 0.991 |
| N | 43 | 43 | 43 | 43 |
| **Number of active pharmaceutical ingredients** | | | | |
| ***Model 1*** | | | | |
| Time (years) | 1.1 (1.0 to 1.2)^*^ | 0.1 (0.08 to 0.12)^*^ | -0.1 (-0.2 to -0.03)^*^ | 1.1 (0.9 to 1.3)^*^ |
| Constant | -2166 (-2419 to -1914)^*^ | -200 (-239 to -162)^*^ | 272 (54 to 490)^*^ | -2095 (-2493 to -1697)^*^ |
| R^2^ | 0.846 | 0.717 | 0.142 | 0.719 |
| R^2^-adjusted | 0.842 | 0.711 | 0.122 | 0.712 |
| ***Model 2*** | | | | |
| 2014–2023 | 9.7 (8.8 to 10.5)^*^ | 1.2 (0.9 to 1.6)^*^ | 4.7 (3.0 to 6.5)^*^ | 15.6 (13.9 to 17.4)^*^ |
| 2007–2013 | 21.4 (20.5 to 22.3)^*^ | 2.0 (1.6 to 2.4)^*^ | -0.2 (-1.7 to 1.2) | 23.2 (21.4 to 24.9)^*^ |
| Constant | 4 | 4.7 (4.4 to 5.0)^*^ | 1.5 (0.1 to 2.9)^*^ | 10.2 (8.7 to 11.7)^*^ |
| R^2^ | 0.970 | 0.731 | 0.673 | 0.960 |
| R^2^-adjusted | 0.968 | 0.718 | 0.656 | 0.958 |
| ***Model 3*** | | | | |
| 2001–2006 | 0.994 | 0.994 | 0.839 | 0.995 |
| 2007–2013 | 0.993 | 0.993 | 0.827 | 0.995 |
| 2014–2023 | 43 | 43 | 43 | 43 |
| R^2^ | 0.994 | 0.994 | 0.839 | 0.995 |
| R^2^-adjusted | 0.993 | 0.993 | 0.827 | 0.995 |
| N | 43 | 43 | 43 | 43 |

^*^P<0.05. (  ) = 95% confidence interval. Time trend coefficients from univariate regressions with Huber-White robust standard errors

**Figure S1:** Gross domestic product deflator for advanced economies, 2001–2024. ⚫ = 100 for January 2024. Linear interpolation between annual exchange rates. Data source: International Monetary Fund World Economic Outlook database, October 2023

**Figure S2:** Normalized prices of active pharmaceutical ingredients in single medicines in the Global Drug Facility Product Catalog, 2001–2024. ⚫ = normalized price. *P<0.05 for price trend. ⚫ = deflated normalized price. ^†^P<0.05 for deflated price trend. First listed price is 100. N = 9–40

**Figure S3:** Normalized prices of active pharmaceutical ingredient combinations in fixed-dose combinations in the Global Drug Facility Product Catalog, 2001–2024. ⚫ = normalized price. *P<0.05 for price trend. ⚫ = deflated normalized price. ^†^P<0.05 for deflated price trend. First listed price is 100. N = 14–40

**Figure S4:** Normalized prices of active pharmaceutical ingredient regimens in patient kits in the Global Drug Facility Product Catalog, 2001–2024. ⚫ = normalized price. *P<0.05 for price trend. ⚫ = deflated normalized price. ^†^P<0.05 for deflated price trend. First listed price is 100. N = 1–37
